# Supplementary figures and images for: Climate suitability predictions for the cultivation of macadamia (Macadamia integrifolia) in Malawi using climate change scenarios
Source: PLoS One. 2021 Sep 9;16(9):e0257007. doi: 10.1371/journal.pone.0257007 (PMC8428786; doi:10.1371/journal.pone.0257007)

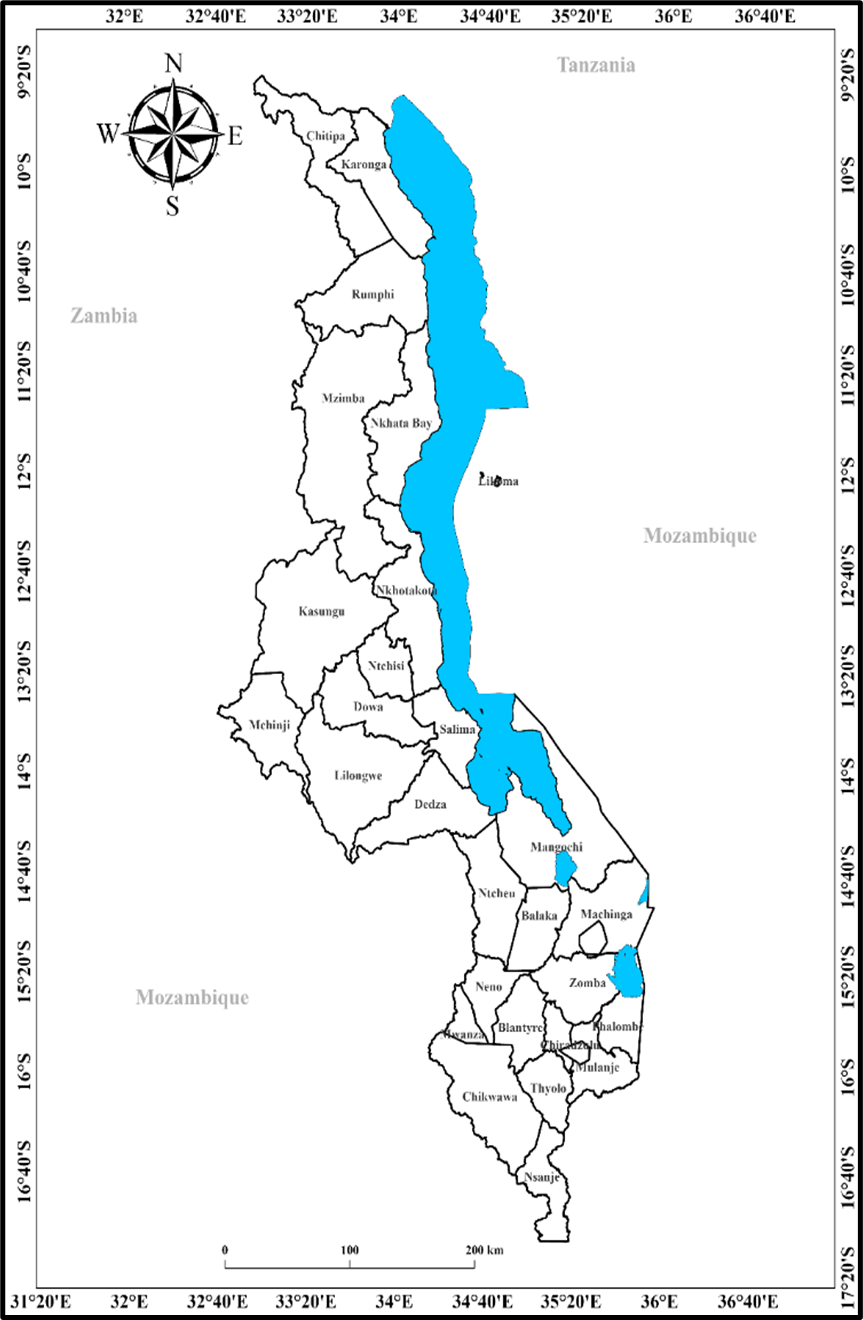

Supplement: S1 Fig — (TIF) [file pone.0257007.s001.tif]
