# Supplementary material for: Climate suitability predictions for the cultivation of macadamia (Macadamia integrifolia) in Malawi using climate change scenarios
Source: PLoS One. 2021 Sep 9;16(9):e0257007. doi: 10.1371/journal.pone.0257007 (PMC8428786; doi:10.1371/journal.pone.0257007)
Supplement: S1 Table — (DOCX) [file pone.0257007.s002.docx]

**Climate suitability predictions for the cultivation of macadamia (*Macadamia integrifolia*) in Malawi using climate change scenarios.**

Emmanuel Junior Zuza^1^*, Kadmiel Maseyk^1^, Shonil A Bhagwat^2^, Kauê de Sousa^3,4^, ^5^Andrew Emmott, ^5^William Rawes, Yoseph Negusse Araya^1^.

**S1 Table**: Suitable climatic conditions for macadamia production in Malawi.

| Description | Category | Adverse | Moderate | Optimal |
| --- | --- | --- | --- | --- |
| Minimum temperature of the coldest month. | T_min_[^o^C] | ≤1 | 1–4 | 5–10 |
| Annual mean temperature. | T_mean_[^o^C] | ≤9 | 10–15 | 16–30 |
| Maximum temperature of the warmest month. | T_max_[^o^C] | ≥36 | 31–35 | 25–30 |
| Annual precipitation. | Prec[mm] | 0–700 & ≥1750 | 900–1000 & 1300–1750 | 1000–1250 |
