# Supplementary material for: Climate suitability predictions for the cultivation of macadamia (Macadamia integrifolia) in Malawi using climate change scenarios
Source: PLoS One. 2021 Sep 9;16(9):e0257007. doi: 10.1371/journal.pone.0257007 (PMC8428786; doi:10.1371/journal.pone.0257007)
Supplement: S2 Table — (DOCX) [file pone.0257007.s003.docx]

**Climate suitability predictions for the cultivation of macadamia (*Macadamia integrifolia*) in Malawi using climate change scenarios.**

Emmanuel Junior Zuza^1^*, Kadmiel Maseyk^1^, Shonil A Bhagwat^2^, Kauê de Sousa^3,4^, ^5^Andrew Emmott, ^5^William Rawes, Yoseph Negusse Araya^1^.

**S2 Table:** Regions and districts in Malawi.

| **Region** | **Name** |
| --- | --- |
| Northern | Chitipa |
|  | Karonga |
|  | Likoma |
|  | Mzimba |
|  | Nkhata Bay |
|  | Rumphi |
| Central | Dedza |
|  | Dowa |
|  | Kasungu |
|  | Lilongwe |
|  | Mchinji |
|  | Nkhotakota |
|  | Ntcheu |
|  | Ntchisi |
|  | Salima |
| Southern | Balaka |
|  | Blantyre |
|  | Chikwawa |
|  | Chiradzulu |
|  | Machinga |
|  | Mangochi |
|  | Mulanje |
|  | Mwanza |
|  | Neno |
|  | Nsanje |
|  | Phalombe |
|  | Thyolo |
|  | Zomba |
