# Supplementary material for: Climate suitability predictions for the cultivation of macadamia (Macadamia integrifolia) in Malawi using climate change scenarios
Source: PLoS One. 2021 Sep 9;16(9):e0257007. doi: 10.1371/journal.pone.0257007 (PMC8428786; doi:10.1371/journal.pone.0257007)
Supplement: S3 Table — (DOCX) [file pone.0257007.s004.docx]

**Climate suitability predictions for the cultivation of macadamia (*Macadamia integrifolia*) in Malawi using climate change scenarios.**

Emmanuel Junior Zuza^1^*, Kadmiel Maseyk^1^, Shonil A Bhagwat^2^, Kauê de Sousa^3,4^, ^5^Andrew Emmott, ^5^William Rawes, Yoseph Negusse Araya^1^.

**S3 Table**: Bioclimatic variables available in WorldClim.

| Covariate | Bioclimatic variable | Unit |
| --- | --- | --- |
| Bio1 | Annual Mean Temperature | ^o^C |
| Bio2 | Mean Diurnal Range (Mean of monthly) | ^o^C |
| Bio3 | Isothermality (BIO2/BIO7) x 100 | - |
| Bio4 | Temperature Seasonality (Std. Dev x 100) | - |
| Bio5 | Max Temperature of Warmest Month | ^o^C |
| Bio6 | Min Temperature of Coldest Month | ^o^C |
| Bio7 | Temperature Annual Range | ^o^C |
| Bio8 | Mean Temperature of Wettest Quarter | ^o^C |
| Bio9 | Mean Temperature of Driest Quarter | ^o^C |
| Bio10 | Mean Temperature of Warmest Quarter | ^o^C |
| Bio11 | Mean Temperature of Coldest Quarter | ^o^C |
| Bio12 | Annual Precipitation | mm |
| Bio13 | Precipitation of Wettest Month | mm |
| Bio14 | Precipitation of Driest Month | mm |
| Bio15 | Precipitation Seasonality (cv x 100) | - |
| Bio16 | Precipitation of Wettest Quarter | mm |
| Bio17 | Precipitation of Driest Quarter | mm |
| Bio18 | Precipitation of Warmest Quarter | mm |
| Bio19 | Precipitation of Coldest Quarter | mm |
