# Supplementary material for: Climate suitability predictions for the cultivation of macadamia (Macadamia integrifolia) in Malawi using climate change scenarios
Source: PLoS One. 2021 Sep 9;16(9):e0257007. doi: 10.1371/journal.pone.0257007 (PMC8428786; doi:10.1371/journal.pone.0257007)
Supplement: S4 Table — (DOCX) [file pone.0257007.s005.docx]

**Climate suitability predictions for the cultivation of macadamia (*Macadamia integrifolia*) in Malawi using climate change scenarios.**

Emmanuel Junior Zuza^1^*, Kadmiel Maseyk^1^, Shonil A Bhagwat^2^, Kauê de Sousa^3,4^, ^5^Andrew Emmott, ^5^William Rawes, Yoseph Negusse Araya^1^.

**S4 Table**. The general circulation models (GCMs) used to obtain climatic variables under scenarios RCP 4.5 and RCP 8.5 in 2050.

| Country | Modelling centre | GCM | Abbreviation |
| --- | --- | --- | --- |
| Australia | Commonwealth Scientific and Industrial Research Organization | ACCESS1-0 AC | AC |
| China | Beijing Climate Center | BCC-CSM1-1 BC | BC |
| USA | National Center for Atmospheric Research | CCSM4 CC | CC |
| France | Centre National de Recherches Météorologiques, Centre Européen de Recherche et de Formation Avancée en Calcul Scientifique | CNRM-CM5 CN | CN |
| USA | Geophysical Fluid Dynamics Laboratory | GFDL-CM3 GF | GF |
| USA | NASA/GISS (Goddard Institute for Space Studies) | GISS-E2-R GS | GS |
| South Korea | National Institute of Meteorological Research, Korea Meteorological Administration | HadGEM2-AO HD | HD |
| UK | Met Office Hadley Centre | HadGEM2-CC HG | HG |
|  |  | HadGEM2-ES HE | HE |
| Russia | Russian Academy of Sciences, Institute of Numerical Mathematics | INMCM4 IN | IN |
| France | Institut Pierre-Simon Laplace | IPSL-CM5A-LR IP | IP |
| Japan | Atmosphere and Ocean Research Institute (The University of Tokyo), National Institute for  Environmental Studies, and Japan Agency for Marine-Earth Science and Technology | MIROC-ESM-CHEM MI | MI |
|  |  | MIROC-ESM MR | MR |
|  |  | MIROC5 MC | MC |
| Germany | Max Planck Institute for Meteorology | MPI-ESM-LR MP | MP |
| Japan | Meteorological Research Institute | MRI-CGCM3 MG | MG |
| Norway | Bjerknes Centre for Climate Research, Norwegian Meteorological Institute | NorESM1-M | NO |
