# Supplementary material for: Climate suitability predictions for the cultivation of macadamia (Macadamia integrifolia) in Malawi using climate change scenarios
Source: PLoS One. 2021 Sep 9;16(9):e0257007. doi: 10.1371/journal.pone.0257007 (PMC8428786; doi:10.1371/journal.pone.0257007)
Supplement: S5 Table — (DOCX) [file pone.0257007.s006.docx]

**Climate suitability predictions for the cultivation of macadamia (*Macadamia integrifolia*) in Malawi using climate change scenarios.**

Emmanuel Junior Zuza^1^*, Kadmiel Maseyk^1^, Shonil A Bhagwat^2^, Kauê de Sousa^3,4^, ^5^Andrew Emmott, ^5^William Rawes, Yoseph Negusse Araya^1^.

**S5 Table**. Characteristics of climate change scenarios by the 2050s (RCPs).

| Scenario | Radioactive forcing (W/m^2^) | GEI concentration by the year 2100 (ppm CO_2_ equivalent) | Temperature change (^o^C) by 2100 | | Greenhouse gas emissions | Agricultural Area | Publication |
| --- | --- | --- | --- | --- | --- | --- | --- |
|  |  |  | Mean | Range |  |  |  |
| RCP 2.6 | 2.6 | ~490 | 1.0 | 0.4–1.6 | Very low. | Medium for cropland and pasture. | (Riahi et al., 2007). |
| RCP 4.5 | 4.5 | ~650 | 1.4 | 0.9–2.0 | Medium-low mitigation (very low baseline). | Very low for both cropland and pasture. | (Fujino et al., 2006). |
| RCP 6.0 | 6.0 | ~850 | 1.3 | 0.8–1.8 | Medium baseline (high mitigation). | Medium for cropland but very low for pasture. | (Clarke et al., 2007). |
| RCP 8.5 | 8.5 | ~1370 | 2.0 | 1.4–2.6 | High baseline. | Medium for cropland but very low for pasture. | (Van Vuuren et al., 2007). |
