# Supplementary material for: Climate suitability predictions for the cultivation of macadamia (Macadamia integrifolia) in Malawi using climate change scenarios
Source: PLoS One. 2021 Sep 9;16(9):e0257007. doi: 10.1371/journal.pone.0257007 (PMC8428786; doi:10.1371/journal.pone.0257007)
Supplement: S6 Table — (DOCX) [file pone.0257007.s007.docx]

**Climate suitability predictions for the cultivation of macadamia (*Macadamia integrifolia*) in Malawi using climate change scenarios.**

Emmanuel Junior Zuza^1^*, Kadmiel Maseyk^1^, Shonil A Bhagwat^2^, Kauê de Sousa^3,4^, ^5^Andrew Emmott, ^5^William Rawes, Yoseph Negusse Araya^1^.

**S6 Table**: Future distribution area of macadamia production in Malawi by 2050.

| RCP | Category | Area (km^2^) | | % changes^a^ | Net change (%)^b^ |
| --- | --- | --- | --- | --- | --- |
| 4.5 | No longer suitable | 17,015 | −18.0 | | −17.8 |
|  | Never suitable | 40,317 | 42.7 | |  |
|  | Remains suitable | 36,910 | 39.1 | |  |
|  | New Areas | 207 | 0.22 | |  |
| 8.5 | No longer suitable | 20,414 | −21.6 | | −21.1 |
|  | Never suitable | 40,047 | 42.4 | |  |
|  | Remains suitable | 33,511 | 35.5 | |  |
|  | New Areas | 476 | 0.5 | |  |

^a^ Percentage of the total land area of Malawi (94,449 km^2^).

^b^ Net change is the balance between colonization and loss (positive net balance indicates an increase in the areas suitable for the species, and negative net balance indicates a decrease in the areas suitable for the species).
