# Supplementary material for: Climate suitability predictions for the cultivation of macadamia (Macadamia integrifolia) in Malawi using climate change scenarios
Source: PLoS One. 2021 Sep 9;16(9):e0257007. doi: 10.1371/journal.pone.0257007 (PMC8428786; doi:10.1371/journal.pone.0257007)
Supplement: S7 Table — (DOCX) [file pone.0257007.s008.docx]

**Climate suitability predictions for the cultivation of macadamia (*Macadamia integrifolia*) in Malawi using climate change scenarios.**

Emmanuel Junior Zuza^1^*, Kadmiel Maseyk^1^, Shonil A Bhagwat^2^, Kauê de Sousa^3,4^, ^5^Andrew Emmott, ^5^William Rawes, Yoseph Negusse Araya^1^.

**S7 Table.** Areas that will remain suitable for macadamia production by the 2050s by region.

| **Region** | **RCP 4.5** | | **RCP 8.5** | |
| --- | --- | --- | --- | --- |
|  | Area (km^2^) | Percentage (%) | Area (km^2^) | Percentage (%) |
| Central | 17,543 | 18.6 | 16,377 | 17.3 |
| Northern | 17,491 | 18.5 | 15,611 | 16.5 |
| Southern | 1,876 | 2.0 | 1,523 | 1.6 |
| Total | 36,910 | 39.1 | 33,511 | 35.5 |
