# Supplementary material for: Climate suitability predictions for the cultivation of macadamia (Macadamia integrifolia) in Malawi using climate change scenarios
Source: PLoS One. 2021 Sep 9;16(9):e0257007. doi: 10.1371/journal.pone.0257007 (PMC8428786; doi:10.1371/journal.pone.0257007)
Supplement: S8 Table — (DOCX) [file pone.0257007.s009.docx]

**Climate suitability predictions for the cultivation of macadamia (*Macadamia integrifolia*) in Malawi using climate change scenarios.**

Emmanuel Junior Zuza^1^*, Kadmiel Maseyk^1^, Shonil A Bhagwat^2^, Kauê de Sousa^3,4^, ^5^Andrew Emmott, ^5^William Rawes, Yoseph Negusse Araya^1^.

**S8 Table.** Summary of news reports about climate change affecting macadamia production worldwide (period 2013-2019).

| Organization | Year | Country | Main report |
| --- | --- | --- | --- |
| [Helvetas](https://www.helvetas.org/en/switzerland/how-you-can-help/follow-us/blog/inclusive-systems/Why-nepali-farmers-decide-to-grow-or-not-grow-nuts) | 2018 | Nepal | Climate change is a challenge in Nepal. Current and projected increases in maximum temperatures and decreases in summer monsoon precipitation may affect macadamia tree growth and yields. |
| [Macadamia Association of Zimbabwe](https://allafrica.com/stories/202001200651.html) | 2020 | Zimbabwe | Macadamia nut farmers experience the poorest harvest ever in 2020 owing to the side effects of climate change. Projected reduced rains and heat waves will likely affect macadamia production in the future within the country. |
| [Agricultural Research Council](https://www.weadapt.org/sites/weadapt.org/files/legacy-new/knowledge-base/files/5370f181a5657504721bd5c21csouth-african-agriculture.pdf) | 2016 | South Africa | Changing climate is slowly altering the South Africa agricultural landscape, and farmers will need to adjust farming practices. Climate suitability studies show that the northern parts of South Africa are shifting towards the south. The Eastern Cape and KwaZulu-Natal areas could become more suitable for growing nuts, like macadamias, while Limpopo could no longer be suitable for nut production by the year 2090. |
| [Macadamia Conservation Trust](https://www.wildmacadamias.org.au/rare-macadamias/threats-facing-wild-macadamias/) | 2017 | Australia | The MCT in Australia reports that macadamia habitat losses and fragmentation are attributed to climate change and urban footprint. New South Wales will lose about 10 % of its macadamia growing areas due to climate change. |
| [The Standard](https://www.standardmedia.co.ke/central/article/2001308253/macadamia-farmers-face-poor-harvest) | 2019 | Kenya | Kenyan macadamia farmers face poor crop harvests during the 2018-19 growing season due to heavy rains during the flowering phase. Interviews with some smallholder farmers reveal that climate changes will likely continue affecting macadamia yields and hence the need for sustainable technologies for adaptation. |
| [International Pacific Research Center](http://iprc.soest.hawaii.edu/newsletters/newsletter_sections/iprc_climate_vol14_1/Climate_change_Hawaii.pdf) | 2014 | Hawai‘i | Climate change is a prominent concern among the general public in Hawai‘i and has received significant official attention within the island nation. Hamilton reports that climate change will complicate agricultural planning in Hawai‘i, especially for perennial tree crops such as macadamia and coffee. |
