# Supplementary material for: Climate suitability predictions for the cultivation of macadamia (Macadamia integrifolia) in Malawi using climate change scenarios
Source: PLoS One. 2021 Sep 9;16(9):e0257007. doi: 10.1371/journal.pone.0257007 (PMC8428786; doi:10.1371/journal.pone.0257007)
Supplement: Attachment — Submitted filename: PONE-D-21-15846_reviewer.pdf [file pone.0257007.s010.pdf]

PLOS ONE

Climatic suitability predictions for the cultivation of macadamia in Malawi using climate change scenarios.

--Manuscript Draft--

**Manuscript Number:**	PONE-D-21-15846
**Article Type:**	Research Article
**Full Title:**	Climatic suitability predictions for the cultivation of macadamia in Malawi using climate change scenarios.
**Short Title:**	Macadamia integrifolia: Climate suitability of Malawi.
**Corresponding Author:**	Emmanuel Junior Zuza, MSc Crop Protection<br>The Open University<br>Milton Keynes, Buckinghamshire UNITED KINGDOM
**Keywords:**	Malawi<br>Macadamia<br>Climate change<br>Ensemble model<br>Climate change scenarios
**Abstract:**	Climate change is altering suitable areas of crop species worldwide, with cascading effects on people and animals reliant upon those crop species as food sources. Macadamia is one of Malawi's most important and profitable crop species. Here, we used an ensemble model approach to determine the current distribution of macadamia producing areas across Malawi in relation to climate. For future distribution of suitable areas, we used the climate outputs of 17 general circulation models (GCM's) based on two climate change scenarios (RCP 4.5 and RCP 8.5). We found that the precipitation of the driest month and isothermality were the climatic variables that strongly influenced macadamia's suitability in Malawi. These climatic requirements were fulfilled across many areas in Malawi under the current conditions. Future projections indicated that large parts of Malawi's macadamia growing regions will remain suitable for macadamia, amounting to 36,910 km2 (39.1%) and 33,511 km2 (35.5%) of land based on RCP 4.5 and RCP 8.5, respectively. Of concern, suitable areas for macadamia production are predicted to shrink by −18% (17,015 km2) and −22% (20,414 km2) based on RCP 4.5 and RCP 8.5, respectively, with much of the suitability shifting northwards. Although a net loss of area suitable for macadamia is predicted, some currently unsuitable areas will become suitable in the future. Notably, suitable areas will increase in Malawi's central and northern regions, while the southern region will lose most of its suitable areas. In conclusion, our study provides critical evidence that climate change will significantly affect the macadamia sub-sector in Malawi. Therefore area-specific adaptation strategies are required to build resilience.
**Order of Authors:**	Emmanuel Junior Zuza, MSc Crop Protection<br>Yoseph Negusse Araya<br>Kadmiel Maseyk<br>Shonil A Bhagwat<br>Kauê de Sousa<br>William Rawes<br>Andrew Emmott
**Additional Information:**	
**Question**	**Response**
**Financial Disclosure**<br><br>Enter a financial disclosure statement that describes the sources of funding for the	YES. The research was funded by The Open University and The UK Research and Innovation through Global Challenges Research Fund (GCRF).

Powered by Editorial Manager® and ProduXion Manager® from Aries Systems Corporation

work included in this submission. Review the submission guidelines for detailed requirements. View published research articles from *PLOS ONE* for specific examples.<br><br>This statement is required for submission and **will appear in the published article** if the submission is accepted. Please make sure it is accurate.<br><br>**Unfunded studies**<br>Enter: *The author(s) received no specific funding for this work.*<br><br>**Funded studies**<br>Enter a statement with the following details:<br>• Initials of the authors who received each award<br>• Grant numbers awarded to each author<br>• The full name of each funder<br>• URL of each funder website<br>• Did the sponsors or funders play any role in the study design, data collection and analysis, decision to publish, or preparation of the manuscript?<br>• **NO** - Include this sentence at the end of your statement: *The funders had no role in study design, data collection and analysis, decision to publish, or preparation of the manuscript.*<br>• **YES** - Specify the role(s) played.<br><br>* typeset	
**Competing Interests**<br><br>Use the instructions below to enter a competing interest statement for this submission. On behalf of all authors, disclose any competing interests that could be perceived to bias this work—acknowledging all financial support and any other relevant financial or non-financial competing interests.<br><br>This statement is required for submission and **will appear in the published article** if the submission is accepted. Please make sure it is accurate and that any funding sources listed in your Funding Information	NO

Powered by Editorial Manager® and ProduXion Manager® from Aries Systems Corporation

later in the submission form are also declared in your Financial Disclosure statement.<br><br>View published research articles from *PLOS ONE* for specific examples.<br><br>**NO authors have competing interests**<br><br>Enter: *The authors have declared that no competing interests exist.*<br><br>**Authors with competing interests**<br><br>Enter competing interest details beginning with this statement:<br><br>*I have read the journal's policy and the authors of this manuscript have the following competing interests: [insert competing interests here]*<br><br>* typeset	
**Ethics Statement**<br><br>Enter an ethics statement for this submission. This statement is required if the study involved:<br><br>• Human participants<br>• Human specimens or tissue<br>• Vertebrate animals or cephalopods<br>• Vertebrate embryos or tissues<br>• Field research<br><br>Write "N/A" if the submission does not require an ethics statement.<br><br>General guidance is provided below. Consult the submission guidelines for detailed instructions. **Make sure that all information entered here is included in the Methods section of the manuscript.**	N/A

**Format for specific study types**<br><br>**Human Subject Research (involving human participants and/or tissue)**<br>• Give the name of the institutional review board or ethics committee that approved the study<br>• Include the approval number and/or a statement indicating approval of this research<br>• Indicate the form of consent obtained (written/oral) or the reason that consent was not obtained (e.g. the data were analyzed anonymously)<br><br>**Animal Research (involving vertebrate animals, embryos or tissues)**<br>• Provide the name of the Institutional Animal Care and Use Committee (IACUC) or other relevant ethics board that reviewed the study protocol, and indicate whether they approved this research or granted a formal waiver of ethical approval<br>• Include an approval number if one was obtained<br>• If the study involved *non-human primates*, add *additional details* about animal welfare and steps taken to ameliorate suffering<br>• If anesthesia, euthanasia, or any kind of animal sacrifice is part of the study, include briefly which substances and/or methods were applied<br><br>**Field Research**<br><br>Include the following details if this study involves the collection of plant, animal, or other materials from a natural setting:<br>• Field permit number<br>• Name of the institution or relevant body that granted permission	
**Data Availability**<br><br>Authors are required to make all data underlying the findings described fully available, without restriction, and from the time of publication. PLOS allows rare exceptions to address legal and ethical concerns. See the PLOS Data Policy and FAQ for detailed information.	Yes - all data are fully available without restriction

Powered by Editorial Manager® and ProduXion Manager® from Aries Systems Corporation

A Data Availability Statement describing where the data can be found is required at submission. Your answers to this question constitute the Data Availability Statement and **will be published in the article**, if accepted.<br><br>**Important:** Stating 'data available on request from the author' is not sufficient. If your data are only available upon request, select 'No' for the first question and explain your exceptional situation in the text box.<br><br>Do the authors confirm that all data underlying the findings described in their manuscript are fully available without restriction?	
**Describe where the data may be found in full sentences. If you are copying our sample text, replace any instances of XXX with the appropriate details.**<br><br>• If the data are **held or will be held in a public repository**, include URLs, accession numbers or DOIs. If this information will only be available after acceptance, indicate this by ticking the box below. For example: *All XXX files are available from the XXX database (accession number(s) XXX, XXX.*).<br>• If the data are all contained **within the manuscript and/or Supporting Information files**, enter the following: *All relevant data are within the manuscript and its Supporting Information files.*<br>• If neither of these applies but you are able to provide **details of access elsewhere**, with or without limitations, please do so. For example:<br><br>*Data cannot be shared publicly because of [XXX]. Data are available from the XXX Institutional Data Access / Ethics Committee (contact via XXX) for researchers who meet the criteria for access to confidential data.*<br><br>*The data underlying the results presented in the study are available from (include the name of the third party*	The data will be uploaded to the Zenodo repository (https://zenodo.org/) and the code on GitHub (https://github.com/EJEYZiE01).

and contact information or URL).<br>• This text is appropriate if the data are owned by a third party and authors do not have permission to share the data.<br><br>* typeset	
Additional data availability information:	Tick here if the URLs/accession numbers/DOIs will be available only after acceptance of the manuscript for publication so that we can ensure their inclusion before publication.

Powered by Editorial Manager® and ProduXion Manager® from Aries Systems Corporation

Cover Letter

Click here to access/download;Manuscript;Zuza et al Suitability models Macadamia-PLOs One_Revision.pdf

Climatic suitability predictions for the cultivation of macadamia in Malawi using climate change scenarios.

Emmanuel Junior Zuza[1]*, Kadmiel Maseyk[1], Shonil A Bhagwat[2], Kauê de Sousa[3,4], [5]Andrew Emmott, [5]William Rawes, Yoseph Negusse Araya[1].

[1]School of Environment, Earth and Ecosystem Sciences, Faculty of Science, Technology, Engineering & Mathematics, The Open University, Gass Building, Walton Hall, Milton Keynes MK7 6AA, The United Kingdom, [2]School of Social Sciences and Global Studies, Faculty of Arts & Social Sciences, The Open University, Gardiner Building, Walton Hall, Milton Keynes MK7 6AA, The United Kingdom, [3]Department of Agricultural Sciences, Faculty of Applied Ecology, Agricultural Sciences and Biotechnology Inland Norway University of Applied Sciences, 2318 Hamar, Norway, [4]Digital Inclusion Area, Biodiversity International, 00054 Maccarese, Italy, [5]The Neno Macadamia Trust, 42 Harpur Street, Bedford MK40 2QT, The United Kingdom.

*Emmanuel.Zuza@open.ac.uk.

Abstract.

Climate change is altering suitable areas of crop species worldwide, with cascading effects on people and animals reliant upon those crop species as food sources. Macadamia is one of Malawi's most important and profitable crop species. Here, we used an ensemble model approach to determine the current distribution of macadamia producing areas across Malawi in relation to climate. For future distribution of suitable areas, we used the climate outputs of 17 general circulation models (GCM's) based on two climate change scenarios (RCP 4.5 and RCP 8.5). We found that the precipitation of the driest month and isothermality were the climatic variables that strongly influenced macadamia's suitability in Malawi. These climatic requirements were fulfilled across many areas in Malawi under the current conditions. Future projections indicated that large parts of Malawi's macadamia growing regions will remain suitable for macadamia,

amounting to 36,910 km^2 (39.1%) and 33,511 km^2 (35.5%) of land based on RCP 4.5 and RCP 8.5, respectively. Of concern, suitable areas for macadamia production are predicted to shrink by −18% (17,015 km^2) and −22% (20,414 km^2) based on RCP 4.5 and RCP 8.5, respectively, with much of the suitability shifting northwards. Although a net loss of area suitable for macadamia is predicted, some currently unsuitable areas will become suitable in the future. Notably, suitable areas will increase in Malawi's central and northern regions, while the southern region will lose most of its suitable areas. In conclusion, our study provides critical evidence that climate change will significantly affect the macadamia sub-sector in Malawi. Therefore area-specific adaptation strategies are required to build resilience.

Keywords: Malawi, macadamia, climate change, ensemble model.

1. Introduction.

Global climate change is having wide-ranging impacts on ecosystems, human health, livelihoods, food security, water supply, and economic growth [1]. These impacts are expected to intensify in direct proportion to the degree of warming. Warming by 2 °C, for example, is expected to increase the number of people exposed to climate-related risks and poverty by several hundred million by the 2050s [1]. This warming poses significant threats to many parts of Africa's current agricultural production systems, particularly among smallholder farming families [2], [3]. Due to the combination of decreased precipitation and increased temperature, Sub-Saharan Africa (SSA) is one of the most vulnerable regions to climate change [4]–[6]. Malawi has been identified as particularly vulnerable to climate change within SSA due to high levels of poverty, limited finances and technology, and a heavy reliance on a predominantly rain-fed agricultural sector for food and nutritional security, economy, and employment [7], [8].

Malawi's agricultural production is already being hampered by climate change. Since the 1960s, average annual temperatures have increased throughout the country by approximately 0.9 °C [6]. Projected temperature data indicates warming throughout Malawi (≥1.5 °C) by the 2050s relative to now. Moreover, all future climate projections (2050–2100) show that Malawi's surface temperatures will rise [9]. Rainfall

predictions for Malawi have higher uncertainty, including variability in both amounts and seasonal patterns [10]. Analysis of 34 climate change models projecting up to the 2090s suggests more frequent dry spells and increases in rainfall intensity [11]. These changes are expected to threaten livelihoods, increase the risk of food insecurity, and negatively affect Malawi's economic growth.

Increased warming and unreliable rainfall within Malawi will impact the landscape and the livelihoods of many rural populations who depend on agricultural activities [9], [12]. Barrueto et al. [13] observed that in Nepal's highlands, increased temperatures led to changes in cropping systems because of an upward shift in perennial tree crops' suitability, including macadamia. Climate change will cause changes in agricultural systems in Malawi, as it has in Nepal. Furthermore, due to Malawi's high deforestation rates, increases in diurnal temperature changes are expected, making crop growth and development more difficult [14]. Consequently, a proper assessment of climate suitability for various crops under current and future climatic conditions is essential for agricultural land use planning in Malawi [12], [15].

Climate suitability is used to assess the relationship between crop distribution and climate factors. Climate suitability, however, only considers climate conditions and does not test socio-economic factors, management practices, and soil physical and chemical factors [16]. The process of climate suitability assessment involves evaluating the impact of climate factors on crop production, defining the climate boundaries for crops before exploring the impacts of climate change [17]. Bock et al. [18] argue that climate suitability assessment should be the first step in agricultural land use planning. For perennial crops, climate suitability assessment is essential because they are long-term investments with high initial costs to establish the crops [19]. Proper planning is, therefore, key to the success of perennial tree production.

On the other hand, Malawi's climate suitability research has primarily focused on cash and staple crops [20]. Cash crops are grown mainly to be sold rather than consumed by the people who grow them [12]. Cashew, coffee, cotton, pulses, sugarcane, tobacco [12], macadamia [20], and tea [21] are all important cash crops in Malawi. Cash crops like macadamia support smallholder producers in two ways: they contribute to food and nutritional security and economic growth since they are used to supplement cereal-based diets and

generate income. Staple crops are eaten regularly and in large amounts, and they make up the majority of the standard diet. In Malawi, currently, the most important staple crop is maize [20].

Malawi's economy and society are built around agriculture [22]. Agricultural operations provide a living for nearly 85% of the country's households [23]. The agricultural sector consists of two distinct sub-sectors: smallholder farmers and commercial estates. About 11% of the rural labor force works on commercial estates to supplement farm income, and around 80% is engaged in the smallholder sub-sector [24]. Smallholder production accounts for 90% of all the country's food (mainly maize) [25]. Despite the contributions of the smallholder sub-sector to Malawi's food security, individually, most smallholders are food insecure on an annual basis [26]. This is due to their reliance on rain-fed agriculture, minimal use of modern farming tools, unpredictable weather conditions, and heavy reliance on monoculture crop production [26], [27]. Moreover, among these smallholder farmers, food security is not permanent, as the transition from food abundance to food shortage can occur in a matter of days when one's income is lost due to bad weather [28], [29].

Malawi's increasing food demand will make it more difficult to meet in the coming decades, as already strained agricultural systems will be threatened by population growth (expected to reach 38.1 million by 2050) and rising incomes, especially among rural communities [29]. As a result, successful agricultural adaptation to changing climate conditions necessitates a thorough understanding of how climate change will affect cultivation patterns and the suitability of various crops. Studies on the projected effects of climate change in Malawi have mainly focused on staple and cash crops, specifically maize and tobacco. Little is known about perennial tree crops, which are essential to addressing the country's future food security uncertainty [30], [31].

An in-depth understanding of how climate change may alter crop suitability is vital for effective agricultural adaptation strategy in Malawi. This is because projected negative effects of climate change may drive smallholder farmers and rural populations to migrate in the future due to decreased productivity and changes in land-use zones. Benson et al. [12] investigated the climate suitability of various important crops grown

in Malawi to formulate mitigation strategies for the projected negative effects of climate change. Despite its growing commercial importance and benefits for food and nutrition security, the suitability of macadamia in Malawi was not assessed in this report.

Macadamia (*Macadamia integrifolia* Maiden & Betche) is an evergreen perennial crop and belongs to the Proteaceae family [32]. It is one of the most highly regarded nuts globally, with a kernel that contains more than 72% oil. Because of their nutritional value, macadamia nuts fetch high market prices, owing to consumer demands [32]. Historically macadamia trees were inter-cropped with coffee, tea, and tung trees in the commercial estates in southern Malawi [20], [33]. Approximately one million macadamia trees have been planted in the commercial estates sub-sector, with over 300,000 trees planted in the smallholder sub-sector, which is expected to increase to over one million in the next decade [20]. Globally, Malawi is the seventh-largest producer of macadamia nuts [20]. Malawi can become the biggest producer of macadamia in the world [20], [33]. This potential is due to the country's ideal altitude and climatic conditions for growth and development, as well as large land pockets among smallholders that allow for expansion.

Macadamia is a lucrative crop among smallholder farmers in Malawi [34]. However, due to macadamia's sensitivity to extreme temperatures and drought, climate change is expected to negatively impact its productivity and suitability in the future [35]. Studies in South Africa found that macadamia yields were lost due to spatio-temporal variability in precipitation and temperature [36]. According to Barrueto et al. [37], macadamia is best suited to areas with annual mean temperatures ranging from 10–30 ^{o}C. Lower day temperatures ($\leq$10 ^{o}C) can be fatal to the crop, and higher temperatures ($\geq$30 ^{o}C) are associated with excessive water loss [38]. Evans [38] suggested a tolerable annual rainfall for macadamia ranging between 1000–1250 mm in Malawi. A detailed summary regarding climatic conditions suitable for macadamia production is given in Supplementary Table S1.

Climate suitability for crop production is critical for developing adaptation and mitigation strategies to the projected negative impacts of climate change on food security. Our study, therefore, aims to assess the suitability for macadamia production under Malawi's current and future climatic conditions. First, we

examine the current spatial distribution of suitable areas for macadamia in Malawi. Then, utilizing bioclimatic variables [39] obtained from downscaled Coupled Model Intercomparison Project 5 (CMIP5) GCM's [40], we model the future distribution of suitable areas for macadamia production based on two emission scenarios (RCP 4.5 and RCP 8.5) of climate change [41]. We focus on climate projections for the 2050s to align with the United Nations framework of global challenges in agriculture and food security [42].

2. Methods.

2.1. Study area.

Malawi falls within the longitudes 30 and 40, and the latitudes −17 and −10 (Supplementary Fig S1). The country spans over 118, 484 km^2, with 94, 449 km^2 (80%) of land area and 24, 035 km^2 (20%) of water surfaces. Because of variations in topography (Fig **1**), parent materials, and management, soil nutritional status varies greatly across the country, particularly among smallholder farmers [15]. As a result, soil characteristics were not taken into account in our analysis.

Fig 1: Geographic location and topography of Malawi based on Shuttle Radar Topography Mission digital elevation model data.

Malawi has a subtropical climate with two distinct seasons: the rainy season, which lasts from November to April and accounts for 90–95% of the annual precipitation, and the pronounced dry season, which lasts from May to October [43]. The topography of Malawi and its proximity to the Indian Ocean and Lake Malawi determine the geographical distribution of temperature and precipitation. The average annual precipitation ranges from 500 mm in low-lying marginal areas to over 3000 mm in high plateau areas [12]. Malawi's mean annual minimum and maximum temperatures are 12 and 32 °C, respectively, with the lowest temperatures in June and July and the highest in October or early November [44]. Fig 2 illustrates the spatial pattern of average annual temperatures (a) and annual precipitation (b).

Fig 2: a) Average annual temperature (ºC) and b) Precipitation (mm) of Malawi based on WorldClim-Global Climate Data.

2.2. Occurrence data.

Data on macadamia tree species' occurrence was collected from smallholder macadamia producing districts during the 2019/20 growing season through a field survey of macadamia farms in Malawi. For our analysis, we only sampled ten-year-old successfully established macadamia orchards under smallholder rainfed conditions. A total of 120 orchards were sampled throughout Malawi. At each farm, the Global Position System (GPS) coordinates (in WGS84 datum) were collected using a global position system (Garmin eTrex Vista® Cx) together with altitude (Fig 3).

Fig 3: Map of Malawi showing macadamia occurrence points and pseudo absent points.

2.3. Climate data.

We used bioclimatic predictors (~1970–2000) from WorldClim data set version 1.4 (http://www.worldclim.org/) at a spatial resolution of 2.5 arc-minute (4.5 km^2 at the equator) to model the current areas suitable for macadamia production in Malawi. Calculated from monthly temperature and precipitation climatologies, these variables describe spatial variations in annual means, seasonality, and extreme/limiting conditions (Supplementary Table S2). We used outputs of these bioclimatic variables from 17 GCM's based on two representative concentration pathways (RCP) of climate change for future predictions [41]. We selected RCP 4.5, which is an optimistic scenario that considers an intermediate GHG concentration and predicts an average increase in temperature by 1.4 °C (0.9–2.0 °C) and RCP 8.5 the most pessimistic scenario, which considers higher GHG emissions concentration with a 1.4–2.6 °C projected increase in mean global temperature by the 2050s (period 2046–2065). To avoid model overfitting, we selected the least correlated variables by applying the variance inflation factor (VIF) analysis and retained those with $VIF < 10$ [45], [46]. Variables with the highest correlation ($VIF \geq 10$) were removed, resulting in eight bioclimatic predictors for our analysis (Table 1).

2.4. Modelling approach.

We modelled the current and future distribution of macadamia species in Malawi based on an ensemble suitability method implemented by the R package BiodiversityR [47]. We used an ensemble modelling technique because it combines predictions from different algorithms and can provide better accuracy in predictions than relying on individual species distribution models [48]. The procedure consisted of four steps.

Firstly, we evaluated the predictive performance accuracy of 18 algorithms of species distributions models (SDM) using a cross-validation technique. Following work by [49] and [50], we divided the occurrence data into two different sets by randomly assigning 70% of the data as a training dataset to fit the model, and the remaining 30% were used as test data to evaluate the model's predictive accuracy. To evaluate the stability of the prediction accuracy, a five-fold (bins) cross-validation replicate was performed in each of the model algorithms as described by [47]. Each SDM algorithm's performance was evaluated from each bin separately after individual algorithms were assessed with data from the other four bins. Cross-validation is used to measure the likely accuracy of model predictions for future outcomes based on each SDM algorithm (probability of a SDM algorithm to correctly predict presence or absence of a species in an area).

The algorithms' performance was measured with the area under the curve (AUC). The AUC value provides a specific measure of model performance, demonstrating the model's ability to locate a randomly selected present observation in a cell of higher probability than a randomly chosen absence observation [48], [51]. We used an AUC value of 0.77 as a threshold to select the best-performing algorithms. Species distribution model algorithms that did not fit this criterion were not used to calculate the ensemble model's suitability [52]. The SDM algorithms used in our analysis were those that can distinguish between suitable and non-suitable areas without needing absence locations [53]. We utilized the presence-only approach because, for agricultural applications of niche models, it is inappropriate to treat areas without current production as entirely unsuitable. Further, it is difficult and rare to determine whether a species is absent in a particular location. Hence, absence data may not represent naturally occurring phenomena [46]. Additionally,

presence-only models can produce reliable predictions from limited presence datasets, meaning that they are robust and a cheaper option for obtaining training datasets [54]. To enhance the ensemble model's predictive ability, we coupled the macadamia occurrence data with 500 randomly pseudo-absence data generated throughout Malawi (Fig 3). We used the pseudo-absence data as opposed to using real absences to avoid underestimation issues [55].

In the second step, we retained only the algorithms that contributed at least 5% to the ensemble suitability (*Se*) [45]. This generated AUC values for each algorithm and the parameters of the response functions that estimated the probability values of species occurrence based on the climate of each grid cell of the study area (Supplementary Table S5). AUC values ranged between 0 and 1, and a value less than 0.5 indicated that the simulated result was worse than random [56]. Classification of model performance estimated by the AUC was: 0.50–0.60 fail; 0.60–0.70 poor; 0.70–0.80 fair; 0.80–0.90 good; 0.90–1.0 excellent [57]. We later combined the results of all the model algorithms by calculating each algorithm's weighted average (weighted by AUC for each model) of each model's probability values to generate the ensemble suitability map. We weighted the AUC values using the following equation:

$$Ensemble\ (S_e) = \frac{\sum_i w_i S_i}{\sum_i w_i} \quad (1)$$

Where the ensemble suitability (S_e) is obtained as a weighted (w) average of suitabilities predicted by the contributing algorithm (S_i).

The third step generated the current distribution maps (probability maps and presence-absence maps) of macadamia under the current climatic conditions. This was based on the weights which were generated during model calibration (cross-validation). To generate the presence-absence layers, we used the maximum sensitivity (true positive$^+$) and maximum specificity (true negative$^-$) approach [58], where we reclassified the distribution maps to binary maps (suitable and unsuitable areas). This method is one of the most reliable for choosing a reclassification method [59], [55], [60]. In this analysis, sensitivity is the proportion of

observed presences correctly predicted and therefore is a measure of omission errors, whereas specificity represents the proportion of correctly predicted absences and thus quantifies commission errors.

To create distribution maps for future bioclimatic conditions, we utilized the same procedure used in the baseline suitability and presence-absence maps but utilized the climate information from each of the 17 future GCMs for RCP 4.5 and RCP 8.5. Since no criteria exist to assess which of the GCM's best predict future climate [61], by incorporating all 17 GCM's, we encompassed all possible changes in the distribution of the macadamia species. To integrate the results of the 17 GCM's presence-absence layers into a single layer, we used the criterion of likelihood scale [59], which requires at least 66% of agreement among GCM's to keep the predicted presence or absence in a given grid cell.

3. Results.

3.1. Factors determining the suitability of macadamia in Malawi.

Precipitation-related variables were the most important in determining suitability for macadamia production in Malawi. Precipitation of the driest month (May–November) was the variable with the greatest relative influence on the suitability for macadamia production. Possibly due to the sensitivity of pod growth during this phase to water scarcity. Among the temperature variables, isothermality (this variable is calculated by dividing mean diurnal temperature range by mean annual temperature range) was the most significant, with a VIF score of 8.95 (Table 1). Our results indicate that annual means have no effect on the suitability for macadamia production in Malawi.

Table 1: Climate variables influencing climate suitability for macadamia production in Malawi.

Covariate	Bioclimatic variable	Unit	VIF Score
Bio14	Precipitation of driest month	mm	9.69
Bio3	Isothermality (BIO2/BIO7) x 100	-	8.95
Bio15	Precipitation seasonality (cv x 100)		8.09
Bio2	Mean diurnal range (Mean of monthly)	°C	8.05
Bio18	Precipitation of warmest quarter	mm	7.01
Bio13	Precipitation of wettest month	mm	6.73
Bio6	Minimum temperature of the coldest month	°C	5.87
Bio4	Temperature seasonality (Std. Dev x 100)	-	4.12

3.2. Current suitability of macadamia in Malawi.

Results of the present (~1970–2000) climate suitability analysis have revealed that 53,925 km^2 (57.4%) of the surface area in Malawi is suitable for macadamia production, while 40,524 km^2 (42.6%) is unsuitable for the crop. Fig 4 depicts the current areas suitable for macadamia production in Malawi. The color gradient variations represent the degree of macadamia suitability per grid cell. Suitability values range from 0 to 1, with values ≥ 0.85 (forest green) indicating highly suitable areas, 0.74–8.4 (jade green) optimal, 0.54–0.74 (mint green) moderate, and ≤ 0.54 (yellow) indicating marginal areas. Optimal suitability was observed across the higher elevated areas and marginal suitability in the lower elevated areas. Suitability peaked in areas around 1000 m.a.s.l., receiving at least 1000–1250 mm of rainfall and temperatures not exceeding 30 °C.

Interestingly, we find optimal suitability in areas where average annual temperatures are too high for macadamia (≥30 °C), specifically around Katunga (Chikwawa), Luchenza (Mulanje), and Nsabwe (Thyolo), all of which are located in southern Malawi. Highly suitable areas were observed in Malawi's mountainous regions with elevation ranging from 1200–1400 m.a.s.l. in some parts of Dowa, Chitipa, Machinga Mulanje, Mzimba, Ntchisi, Nkhatabay, Rumphi, and Thyolo. Areas with moderate suitability were predicted in the mid-hills between 950–1000 m.a.s.l. in Blantyre, Chiradzulu, Dedza, Kasungu, Lilongwe, Mchinji, Mwanza, Neno, Ntcheu, and Zomba districts. Marginally suitable areas were found to be in the lower

elevated (≤ 900 m.a.s.l) parts of Malawi. As expected, our predictions suggest that the current distribution of climatically suitable areas for macadamia largely overlapped the areas where macadamia are grown in Malawi. Though, these areas are also used for the production of other crops, particularly annuals.

Fig 4: Current suitability for macadamia production in Malawi. The model results were exported into Arc GIS Pro Software Version 2.5 to generate the map in this figure.

3.3. Gains and losses of macadamia suitability under future projections in Malawi.

Under both emission scenarios used in this analysis, the extent of suitable areas for macadamia production is expected to decrease in the future. Our findings show a net loss of –18% and –22% of suitable land for macadamia production under RCP 4.5 and RCP 8.5, respectively (Fig 5, Table 2). This translates to 17,015 km^2 (RCP 4.5) and 20,414 km^2 (RCP 8.5) of Malawi's total cultivatable surface area. Lower altitudes (500–1000 m.a.s.l.) will experience the greatest decline in suitability due to projected heatwaves, flooding, and droughts linked to the El Niño Southern Oscillation. These losses will be more pronounced in Malawi's southern region areas, especially those along the Shire valley. Thyolo district, which is currently the country's most productive and biggest macadamia growing area, is projected to suffer significant reductions in suitable areas for macadamia production due to climate change. Therefore, investment in irrigation infrastructure, both now and in the future, remains a viable option for continuing macadamia production in vulnerable areas in Malawi.

Fig 5: Shifts in macadamia suitability due to climate change by 2050 (a) RCP 4.5 (b) RCP 8.5. The model results were exported into Arc GIS Pro Software Version 2.5 to generate the map in this figure.

Surface gains for macadamia suitability under future climate projections are described in Fig 5, Table 2. Both scenarios show that a large fraction of suitable areas for macadamia production will remain unchanged, particularly in Malawi's central and northern regions. Approximately 36,910 km^2 and 33,511 km^2 of Malawi's surface areas are projected to remain unchanged under RCP 4.5 and RCP 8.5, respectively. The optimistic scenario (RCP 4.5) predicted an average gain (newer areas) of +0.22% of Malawi's surface area,

which equates to approximately 207 km^2 of potentially suitable land for macadamia. Under RCP 8.5 scenario, newer areas are projected to account for +0.5% of the land, translating to 476 km^2 of Malawi's total surface area. We observe that projected newer areas will approximately double RCP 4.5 under RCP 8.5 (0.28%) but still within model uncertainty of no detectable change. This is because some currently very cold areas will become suitable due to the increased warming predicted by RCP 8.5. Based on both emission scenarios, newer areas are expected to occur in Dedza (Mua and Chipansi), Mangochi (Namwera and Chaponda), Ntcheu (Tsangano and Bonga), Rumphi (Nyika) districts. However, these only apply to a small portion of the country and cannot compensate for the decrease in suitability in the country.

Table 2: Future distribution area of macadamia production in Malawi by 2050.

RCP	Category	Area (km^2)	% changes[a]	Net change (%)[b]
4.5	No longer suitable	17,015	–18.0	–17.8
	Never suitable	40,317	42.7	
	Remains suitable	36,910	39.1	
	New Areas	207	0.22	
8.5	No longer suitable	20,414	–21.6	–21.1
	Never suitable	40,047	42.4	
	Remains suitable	33,511	35.5	
	New Areas	476	0.5	

[a] Percentage of the total land area of Malawi (94,449 km^2).

[b] Net change is the balance between colonization and loss (positive net balance indicates an increase in the areas suitable for the species, and negative net balance indicates a decrease in the areas suitable for the species).

4. Discussion.

Precipitation and temperature have been identified as critical factors influencing crop growth and yields worldwide [46]. We find that in Malawi, suitability for macadamia is influenced by precipitation and temperature and seasonal variations of these two factors, rather than the annual means, confirming previous findings [38]. However, climatic variables identified in our study are different from climate indicators for macadamia on a global scale [38]. Conversely, in Nepal, temperature-based factors were identified primarily as determinants for the suitability for macadamia [37]. Chemura et al. [54] found that such variations are explained by differences in scale and geography, indicating that local and regional factors can

influence the potential for macadamia. Thus explaining our findings where precipitation-based factors have been identified as more important in determining the suitability for macadamia, verifying zoning studies for macadamia production done for the country [62]. Consequently, projections that climate change will reduce rainfall amounts making its distribution unreliable in many parts of Malawi [10], will drive many areas out of macadamia production. In this regard, farmers are encouraged to adopt moisture conservation measures (mulching, rainwater harvesting, box ridging, and basins) and possibly develop irrigation infrastructure to match the water requirements for macadamia growth and development annually.

Isothermality was the second most important factor determining the suitability for macadamia in Malawi. Our findings suggest that the large fluctuations in day and night temperatures and increased warming are responsible for the marginal suitability for macadamia, notably along the lakeshore and Shire valley. Optimal suitability was observed in intermediate to upper elevated areas that experience cooler temperatures, especially at night. Consequently, projections that climate change will increase the number of hot ($\geq 30\,^{o}$C) days (30.5 days per year) and hot nights (40 days per year) will certainly reduce the number of suitable areas for macadamia production in Malawi [11]. Therefore, trees currently grown in the hotter areas will require sufficient water availability to cater to the water lost through evapotranspiration. High day and nighttime temperatures have been linked to a reduction in suitable areas for macadamia in Australia [38], Nepal [37], and South Africa [36], findings that are consistent with our current findings in Malawi.

Malawi is very vulnerable to climate change compared to other countries in SSA due to its geographic location and socio-economic status [7], [8]. Our results show that extensive areas in Malawi under the current climatic conditions are suitable for macadamia production. Moreover, our outcomes show the suitability for macadamia in Malawi's south-eastern parts beyond the current reported production areas. This is expected as the suitability maps capture the potential production areas, some of which have not yet been translated to realized areas [46]. However, areas of Malawi already fall outside the prescribed optimal range for macadamia production, which may be attributed to the increase in annual mean temperatures (0.9 ^{o}C) and overall drying recorded in the past five decades [6], [63], [64].

Due to the projected temperature increases and changing rainfall patterns, the suitability for macadamia production in Malawi is likely to decline in the 2050s and is expected to shift northwards. Areas in southern Malawi will be the most affected, with the central and northern highlands even improving capacity to sustain macadamia production in areas where this is impossible due to environmental constraints. Other authors have predicted similar effects of climate change on the suitability for macadamia production globally. Barrueto et al. [37] reported an upward shift in suitable areas for macadamia production in Nepal due to climate warming. This shift resulted in losses in suitable areas for macadamia production in This shift resulted in losses in suitable areas for macadamia production in Nepal's lowlands and gains in suitable areas for the highlands, which is consistent with our findings.

Our results, therefore, show the sensitivity of macadamia to variations in environmental conditions. Thus, in areas where there are no predicted changes in the suitability for macadamia, farmers can continue planting their macadamia trees. However, both research and field-based evidence from discussions with farmers show that climate-related changes are already occurring and affecting the suitability for macadamia production in Malawi. Farmers are, therefore, encouraged to start implementing adaptation measures such as the use of improved macadamia varieties, agroforestry, intercropping, water conservation, and irrigation for long-term and sustainable macadamia production. Nevertheless, these suitability changes are predicted to occur over the next 30 years, so these will mostly impact the next generation of macadamia farmers. Therefore, there is still time for adaptation.

4.1. Applicability and potential limitations of this study.

Species distribution modelling is founded on assumptions intrinsic in the models, some of which cannot be tested [65], [66]. Although this study's findings can be considered robust, several issues should be considered in the interpretation and application of the results. Though we identified areas as suitable for macadamia production based on environmental factors, however on the ground, this may not directly translate to the size of the arable land. Other physical and socio-economic factors (including the gender and age of the smallholder farmers, availability of agricultural advisory services, and market availability) that

determine suitability are difficult to capture in this type of modelling and should be considered in applying model results. Due to these challenges, the authenticity of models in making predictions is questioned [76–77], but modeling remains an important tool for future planning purposes [60], [78], [79]. Therefore, there is a need for a thorough evaluation of adaptation approaches suggested for smallholder macadamia farmers, as these may be different from those utilized by commercial growers.

5. Conclusions.

This study utilized an ensemble model to identify current and future suitability for macadamia production in Malawi. Three important conclusions can be drawn from the study results. First, precipitation factors are the most important determinants for macadamia suitability in Malawi. Second, the current and future areas identified as suitable for macadamia production exist on agricultural land currently used to grow other crops. As a result, we propose promoting macadamia intercrops and agroforestry as a climate change adaptation strategy. Third, the extent of suitable areas for macadamia production in Malawi is projected to decrease under both emission scenarios utilized in this analysis, and the most vulnerable areas are those in southern Malawi. We conclude that the macadamia sector faces production risks from climate change, but there are opportunities for adaptation strategies to build a resilient sector in Malawi.

Acknowledgments.

The authors are grateful to the Open University and the UK Research and Innovation through Global Challenges Research Fund (GCRF) project for funding and academic guidance. Thanks go to Dr. Phillip Holden, Dr. Julia Cooke and the R-Club at The Open University, UK, Prof. Rick Brandenburg, North Carolina State University, USA, Dr. Abel Chemura, Potsdam Institute for Climate Impact Research (PIK), German, Dr. Michael G. Chipeta, Oxford University, Dr. Edith B. Milanzi, MRC Clinical Trials, University College London, the Neno Macadamia Trust, UK and Highlands Macadamia Cooperative Union Limited (HIMACUL) smallholder farmers, Malawi. We further express our gratitude to Mr. Ken Mkangala and

Nicholas Evans for their constructive comments and feedback on the state of macadamia production in Malawi. However, mistakes and omissions are our responsibility.

Competing Interests.

The authors declare no conflict of interests.

Author contributions.

Conceptualization: Emmanuel Junior Zuza, Yoseph N. Araya, Kadmiel Maseyk, Shonil Bhagwat, Andrew Emmott.

Data curation : Emmanuel Junior Zuza, Kauê de Sousa.

Formal analysis : Emmanuel Junior Zuza, Kauê de Sousa.

Methodology : Emmanuel Junior Zuza, Kauê de Sousa, Kadmiel Maseyk, Yoseph Araya, Shonil Bhagwat.

Software : Emmanuel Junior Zuza, Kauê de Sousa.

Supervision : Yoseph N. Araya, Kadmiel Maseyk, Shonil Bhagwat, Andrew Emmott.

Validation : Emmanuel Junior Zuza, Yoseph N. Araya, Kauê de Sousa, Kadmiel Maseyk, Shonil Bhagwat, Andrew Emmott.

Visualization: Emmanuel Junior Zuza, Kauê de Sousa, Yoseph N. Araya, Shonil Bhagwat, William Rawes.

Writing – original draft : Emmanuel Junior Zuza, Yoseph Araya, Kadmiel Maseyk, Shonil Bhagwat.

Writing – Review and Editing : Emmanuel Junior Zuza, Kauê de Sousa, Kadmiel Maseyk, Shonil Bhagwat, Yoseph N. Araya, Andrew Emmott, William Rawes.

References

[1] Xu C, Kohler TA, Lenton TM, Svenning JC, Scheffer M. Future of the human climate niche. *Proc. Natl. Acad. Sci. USA.* 2020; 117:21. https://doi.org/10.1073/pnas.1910114117.

[2] Sultan B, Defrance D, Iizumi T. Evidence of crop production losses in West Africa due to historical global warming in two crop models. *Sci. Rep.* 2019. pp. 1–15. https//doi.org/10.1038/s41598-019-49167-0.

[3] Woetzel J, Pinner D, Samandari H, Engel H, McCullough R, Melzer T, Boettiger S. How will African farmers adjust to changing patterns of precipitation. *McKinsey Global Institute,* Chicago, USA. 2020.

[4] Niang I, Rupper OC, Abdrado MA, Essel A, Lennard C, Padgham J, Urquhart P. Africa. In: Climate Change 2014: Impacts, Adaptation, and Vulnerability. Part B: Regional Aspects of Working Group II to the Fifth Assessment Report of the Intergovernmental Panel on Climate Change [Barros VR, Field CB, Dokken DJ, Mastrandrea MD, Mach KJ, Bilir TE, Chatterjee M, Ebi KL, Estrada YO, Genova RC, Girma B, Kissel ES, Levy AN, MacCranken S, Mastrandrea PR, White LL (eds)]. *Cambridge University Press*, *Cambridge*, The United Kingdom and New York, NY, USA. 2014. pp. 1199–1266. https//doi.org/10.1017/CBO9781107415386.002.

[5] Rivrud IM, Meisingset EL, Loe LE, Mysterud A. Future suitability of habitat in a migratory ungulate under climate change. *Proceedings. Biol. Sci.* 2019; 286:20190442. https//doi.org/10.1098/rspb.2019.0442.

[6] Dougill AJ, Mkwambisi D, Vincent K, Archer E, Bhave A, Malinga RH, Mataya DC. How can we improve the use of information for a climate-resilient Malawi? 2019. Available online: www.futureclimateafrica.org (accessed on 7 September 2019).

[7] Mataya DC, Vincent K, Dougill AJ. How can we effectively build capacity to adapt to climate change? Insights from Malawi. *Clim. Dev.* 2019. https//doi.org/10.1080/17565529.2019.1694480.

[8] Warnatzsch EA, Reay DS. Assessing climate change projections and impacts on Central Malawi's maize yield: The risk of maladaptation. *Sci. Total Environ.* 2020: 711. 134845, https//doi.org/10.1016/j.scitotenv.2019.134845.

[9] Mittal D, Vincent N, Conway K, van Garderen D, Pardoe EA, Todd J, Washington M, Siderius R, Mkwambisi D. Future climate projections for Malawi. 2017. Available online: www.futureclimateafrica.org (accessed on 10 January 2021).

[10] Warnatzsch EA, Reay DS. Temperature and precipitation change in Malawi: Evaluation of CORDEX-Africa climate simulations for climate change impact assessments and adaptation planning. *Sci. Total Environ.* 2019; 654: 378–392. https//doi.org/10.1016/j.scitotenv.2018.11.098.

[11] The World Bank, "Malawi Country Environmental Analysis. The World Bank, Washington DC, USA. 2019. Available: https://dx.doi.org/10.1016/j.cirp.2016.06.001%0Ahttp://dx.doi.org/10.1016/j.powtec.2016.12.055%0Ahttps://doi.org/10.1016/j.ijfatigue.2019.02.006%0Ahttps://doi.org/10.1016/j.matlet.2019.04.024%0Ahttps://doi.org/10.1016/j.matlet.2019.127252%0Ahttp://dx.doi.o.

[12] Benson T, Mabiso A, Nankhuni F. Detailed crop suitability maps and an agricultural zonation scheme for Malawi. Michigan State University, Michigan, USA. 2016. Available: http://dec.usaid.gov/.

[13] Barrueto AK, Niraula R, Merz J, Pokharel B, Hammer T. Climatic suitability predictions for the cultivation of macadamia and walnuts in existing land-use zones and forest management regimes

under climate change scenarios: addressing food security in the mid-hills of Nepal. *For. Trees Livelihoods*. 2018; 27: 86–102. https//doi.org/10.1080/14728028.2018.1438930.

[14] Chibwana GA, Mapemba LD, Masikat P, Homann-Kee ST, Crespo O, Bandason E. Modeling potential impacts of future climate change in Mzimba district, Malawi, 2040-2070. An integrated biophysical and economic Modeling approach. *International Food Policy Research Institute*, Washington DC, USA. 2014. pp 17.

[15] Li G, Messina JP, Peter BG, Snapp SS. Mapping land suitability for agriculture in Malawi. *L. Degrad. Dev.* 2017; 28: 2001–2016. https//doi.org/10.1002/ldr.2723.

[16] Wang C, Shi X, Liu J, Zhao J, Bo X, Chen F. Chu Q. Interdecadal variation of potato climate suitability in China. *Agric. Ecosyst. Environ.* 2021; 28: 2001–2016. https//doi.org/10.1002/ldr.2723.

[17] Taghizadeh-Mehrjardi R, Nabiollahi K, Rasoli L, Kerry R, Scholten T. Land suitability assessment and agricultural production sustainability using machine learning models. *Agronomy*. 2020; 10: 1–20. https//doi.org/10.3390/agronomy10040573.

[18] Bock M, Gasser PY, Pettapiece WW, Brierley AJ, Bootsma A, Schut P, Neilsen D, Smith S. The land suitability rating system is a spatial planning tool to assess crop suitability in Canada. *Front. Environ. Sci.* 2018; 6: 1–16 https//doi.org/10.3389/fenvs.2018.00077.

[19] Skevas T, Swinton MS, Tanner S, Sanford G, Thelen K.D. Investment risk in bioenergy crops. *Bioenergy*. 2016; 8: 1162–1177. https//doi.org/10.1111/gcbb.12320.

[20] Zuza EJ, Bhagwat S, Emmott A, Rawes W, Maseyk K, Araya YN. Review of Macadamia Production in Malawi: Focusing on What, Where, How Much Is Produced and Major Constraints. *Agriculture*. 2021. 11; 2:152. https://doi.org/10.3390/agriculture11020152.

[21] International Center for Tropical Agriculture (CIAT). Identification of suitable tea Growing areas in Malawi under climate change scenarios. 2017. [Online]. Available: https://utz.org/wp-content/uploads/2017/12/CIAT-report-climate-impact-tea-production-Malawi.pdf.

[22] van Vagt D. Participatory approaches to diversification and intensification of crop production on smallholder farms in Malawi. *The Wageningen University*, Wageningen, Netherlands. Ph.D. Theses. 2018. https://doi.org/https://doi.org/10.18174/456315.

[23] Bezner Kerr R, Nyantakyi-Frimpong H, Dakishoni L, Lupafya E, Shumba L, Luginaah I, Snapp SS. Knowledge politics in participatory climate change adaptation research on agroecology in Malawi. *Ren. Agr and Fd Syst*. 2018; 33: 238–251. https://doi.org/10.1017/S1742170518000017.

[24] The Government of Malawi. National Agricultural Investment Plan (NAIP), 2011-2014. *The Government of Malawi*, Lilongwe, Malawi. 2018. pp 40.

[25] Gondwe S, Kasiya S, Maulidi F, Timanyechi G. Assessment of youth employment initiatives in Malawi : Implementation realities and policy perspectives. *FARA Research Report*. 2020. 5:6. pp. 32.

[26] Sutcliffe CA. Adoption of improved maize cultivars for climate vulnerability reduction in Malawi. *The University of Leeds*, Leeds, The United Kingdom. Ph.D. Theses. 2014.

[27] Chingala G, Mapiye C, Raffrenato E, Hoffman L, Dzama K. Determinants of smallholder farmers' perceptions of the impact of climate change on beef production in Malawi. *Clim. Change*. 2017; 142: 129–141. https//doi.org/10.1007/s10584-017-1924-1.

[28] The Malawi Vulnerability Assessment Committee. Food security forecast for the 2018/19 Consumption Year. 2018. [Online]. Available: https://reliefweb.int/report/malawi/malawi-vulnerability-assessment-committee-mvac-bulletin-no-1518-volume-1-food-security.

[29] Goodman KD, Dahir PS, Singh AL. The other way Covid will kill: Hunger. *The New York Times*, New York, NY, USA, Sep. 11, 2020.

[30] Snapp S, Jayne TS, Mhango W, Benson T, Ricker-Gilbert J. Maize yield response to nitrogen in Malawi's smallholder production systems. *Natl. Symp.* 2014. pp. 13. [Online]. Available:http://ebrary.ifpri.org/utils/getfile/collection/p15738coll2/id/128436/filename/128647.pdf%0Ahttps://ageconsearch.umn.edu/record/188570.

[31] Coulibaly JY, Mbow C, Sileshi GW, Beedy T, Kundhlande G, Musau J. Mapping vulnerability to climate change in Malawi: Spatial and social differentiation in the Shire River Basin. *Am. J. Clim. Chang.* 2015; 4: 282–294. https//doi.org/10.4236/ajcc.2015.43023.

[32] Alam M, Hardner C, Nock C, O'Connor K, Topp B. Historical and molecular evidence of the genetic identity of macadamia cultivars HAES 741 and HAES 660. *HortScience.* 2019; 54: 616–620. https//doi.org/10.21273/HORTSCI13318-18.

[33] Parshotam A. Cultivating smallholder inclusion in southern Africa's Macadamia Nut Value Chains. *South African Institute of International Affairs*, Pretoria, South Africa. 2018.

[34] Toit JP, Nankhuni FJ, Kanyamuka JS. Can Malawi increase its share of the global macadamia market? Opportunities and threats to the expansion of Malawi's macadamia Industry. *Michigan State University*, Michigan, USA. 2017. pp. 20.

[35] Moncur T, Stephenson MW, Trochoulia RA. Floral development of *Macadamia integrifolia* Maiden and Betche under Australian conditions. *Sci. Hortic.* 1985; 27: 87–96.

[36] Britz A. Studies on Macadamia nut quality. The University of Stellenbosch, Stellenbosch, South Africa, MSc Theses. 2015.

[37] Barrueto AK, Merz J, Hodel E, Eckert S. The suitability of Macadamia and Juglans for cultivation in Nepal: an assessment based on spatial probability modelling using climate scenarios and in situ data. *Reg. Environ. Chang.* 2018; 18: 859–871. https//doi.org/10.1007/s10113-017-1225-2.

[38] Nagao MA, Ho-a EB, Yoshimoto JM. Relationship between vegetative flushing and flowering of macadamia. *HortScience*. 1994; 27: 669–669. https//doi.org/10.21273/hortsci.27.6.669g.

[39] Hijmans RJ, Cameron SE, Parra JL, Jones PG, Jarvis A.. Very high resolution interpolated climate

surfaces for global land areas. *Int. J. Climatol.* 2005; 25: 1965–1978. https//doi.org/10.1002/joc.1276.

[40] IPCC. Climate Change 2007: The Physical Science Basis. Contribution of Working Group I to the Fourth Assessment Report of the Intergovernmental Panel on Climate Change [Solomon, S., D. Qin, M. Manning, Z. Chen, M. Marquis, K.B. Averyt, M. Tignor, and H.L. Miller. *Cambridge University Press*, Cambridge, The United Kingdom and New York, NY, USA. 2007. pp 996.

[41] van Vuuren DP, Edmonds J, Kainuma M, Riahi K, Thomson A, Hibbard K, Hurtt GC, Kram T, Krey V, Lamarque JF, Masui T, Meinshausen M, Nakicenovic N, Smith SJ, Rose SK. The representative concentration pathways: An overview. *Clim. Change*. 2011; 109: 5–31. https//doi.org/10.1007/s10584-011-0148-z.

[42] Lipper L, Thornton P, Campbell BM, Baedeker T, Braimoh A, Bwalya M, Caron P, Cattaneo A, Garrity D, Henry K, Hottle R, Jackson L, Jarvis A, Kossam F, Mann W, McCarthy N, Meybeck A, Neufeldt H, Remington T, Torquebiau EF. Climate-smart agriculture for food security. *Nat. Clim. Chang.* 2014; 4: 1068–1072. https//doi.org/10.1038/nclimate2437.

[43] The Government of Malawi. Annual Economic Report Annual. The Government of Malawi, Lilongwe, Malawi. 2020. pp 301.

[44] Mutegi J, Kabambe V, Zingore S, Harawa R, Wairegi L. The status of fertilizer recommendation in Malawi: Gaps, challenges, and opportunities. Soil Health Consortium of Malawi. 2015. pp 56.

[45] Ranjitkar S, Sujakhu NM, Merz J, Kindt R, Xu J, Matin MA, Ali M, Zomer RJ. Suitability analysis and projected climate change impact on banana and coffee production zones in Nepal. *PLoS One*. 2016; 11. https//doi.org/10.1371/journal.pone.0163916.

[46] Chemura A. Modelling spatial variability of coffee (*Coffea arabica* L) crop condition with multispectral remote sensing data. The University of KwaZulu-Natal, KwaZulu, South Africa, Ph.D. Theses. 2017.

[47] Kindt R. Ensemble species distribution modelling with transformed suitability values. *Environ. Model. Softw.* 2018; 100: 136–145. https//doi.org/10.1016/j.envsoft.2017.11.009.

[48] de Sousa K, Solberg SØ. Conservation gaps in traditional vegetables native to Europe and Fennoscandia. *Agriculture*. 2020. 10: 8. 340. https//doi.org/10.3390/agriculture10080340.

[49] Brotons L, Thuiller W, Araújo MB, Hirzel AH. Presence-absence versus presence-only modelling methods for predicting bird habitat suitability," *Ecography (Cop.).* 2004; 27: 437–448. https//doi.org/10.1111/j.0906-7590.2004.03764.x.

[50] Thuiller W, Lavergne S, Roquet C, Boulangeat I, Lafourcade B, Araujo M. B. Consequences of climate change on the tree of life in Europe. *Nature.* 2011; 470: 531–534. https//doi.org/10.1038/nature09705.

[51] Rabara RC, Sotto RC, Salas EAL. Species distribution modeling and phenotypic diversity reveals a

collection gap in the *Musa balbisiana* germplasm conservation in the Philippines. *Asian J. Agric.* 2020; 4 (2): 60–71. https//doi.org/10.13057/asianjagric/g040203.

[52] Jiménez-Valverde A. Threshold-dependence as a desirable attribute for discrimination assessment: implications for the evaluation of species distribution models. *Biodivers Conserv*. 2014; 23: 369–385. htts//doi.org/10.1007/s10531-013-0606-1.

[53] de Sousa K, van Zonneveld M, Imbach P, Casanoves F, Kindt R, Ordoñez JC. Suitability of key Central American agroforestry species under future climates: An atlas. *ICRAF Occasional Paper* no. 26. Turrialba, Costa Rica. 2017.

[54] Chemura A, Mudereri BT, Yalew AW, Gornott C. Climate change and specialty coffee potential in Ethiopia. *Sci. Rep.* 2021; 1–13. htts//doi.org/10.1038/s41598-021-87647-4.

[55] Gama M, Crespo D, Dolbeth M, Anastácio P. Predicting global habitat suitability for *Corbicula fluminea* using species distribution models: The importance of different environmental datasets. *Ecol. Modell.* 2016; 319: 163–169. https//doi.org/10.1016/j.ecolmodel.2015.06.001.

[56] de Sousa K. Agrobiodiversity and climate adaptation: insights for risk management in small-scale farming. *Inland Norway University of Applied Sciences*. 2020: Ph.D. Theses.

[57] Jorcin P, Barthe L, Berroneau M, Dore F, Geniez P, Grillet P, Kabouch B, Movia A, Naimi B, Pottier G, Thirion JM, Cheylan M. Modelling the distribution of the Ocellated Lizard in France: Implications for conservation," *Amphib. Reptil. Conserv.* 2019; 13: 276–298, 2019.

[58] Liu C, White M, Newell G. Selecting thresholds for the prediction of species occurrence with presence-only data. *J. Biogeogr.* 2013; 40:778–789. https//doi.org/10.1111/jbi.12058.

[59] de Sousa K, van Zonneveld M, Holmgren M, Kindt R, Ordoñez JC. The future of coffee and cocoa agroforestry in a warmer Mesoamerica. *Sci. Rep.* 2019; 9: 1–9. https//doi.org/10.1038/s41598-019-45491-7.

[60] Domisch S, Araújo MB, Bonada N, Pauls SU, Jähnig SC, Haase P. Modelling distribution in European stream macroinvertebrates under future climates. Glob. Chang. Biol. 2013; 19: 752–762. https//doi.org/10.1111/gcb.12107.

[61] Mastrandrea MD, Field CB., Stocker TF, Edenhofer O, Ebi KL, Frame DJ, Held H, Kriegler E, Mach KJ, Matschoss PR, Plattner GK, Yohe GW, Zwiers FW. Guidance note for lead authors of the IPCC Fifth Assessment Report on Consistent Treatment of Uncertainties. 2010. [Online]. Available: http://www.ipcc.ch.

[62] Evans N. Suitability Mapping of the Malawi macadamia industry. *Consultation Report*. 2008.

[63] Stevens T, Madani K. Future climate impacts on maize farming and food security in Malawi. *Sci. Rep.* 2016; 6. https//doi.org/10.1038/srep36241.

[64] Mittal D, Vincent N, Conway K, van Garderen D, Pardoe E.A, Todd J, Washington M, Siderius R, Mkwambisi D. Future climate projections for Malawi. 2017: Available online:

www.futureclimateafrica.org (accessed on 10 January 2021).

[65] Mittal D, Vincent N, Conway K, van Garderen D, Pardoe E.A, Todd J, Washington M, Siderius R, Mkwambisi D. Future climate projections for Malawi. 2017: Annex. Available online: www.futureclimateafrica.org (accessed on 10 January 2021).

[66] Kutywayo D, Chemura A, Kusena W, Chidoko P, Mahoya C. The impact of climate change on the potential distribution of agricultural pests: The case of the coffee white stem borer (*Monochamus leuconotus* P.) in Zimbabwe. *PLoS One*. 2013; 8. https//doi.org/10.1371/journal.pone.0073432.

[67] Heikkinen RK, Luoto M, Araújo MB, Virkkala R, Thuiller W, Sykes MT. Methods and uncertainties in bioclimatic envelope modelling under climate change. *Prog. Phys. Geogr*. 2006; 30: 751–777. https//doi.org/10.1177/0309133306071957.

[68] Ranjitkar S, Sujakhu NM, Lu Y, Wang Q, Wang M, He J, Mortimer PE, Xu J, Kindt R, Zomer R. Climate modelling for agroforestry species selection in Yunnan Province, China. *Environ. Model. Softw*. 2016; 75: 263–272. https//doi.org//10.1016/j.envsoft.2015.10.027.

[69] Pereira HM, Leadley PW, Proenca V, Alkemade R, Scharlemann JPW, Fernandez-Manjarres JF, Araujo MB, Balvanera P, Biggs R, Cheung WWL, Chini L, Cooper HD, Gilman EL, Guenette S, Hurtt GC, Huntington HP, Mace GM, Oberdorff T, Revenga C. Scenarios for Global Biodiversity in the 21st Century: Supporting information. *Science*. 2010; 80: 496–1502, 2010, [Online]. Available: http://science.sciencemag.org/content/sci/330/6010/1496.full.pdf%0Ahttp://api.iucnredlist.org/go/panthera-leo.

Figure 1

Click here to access/download;Figure;Elev_MW.tif

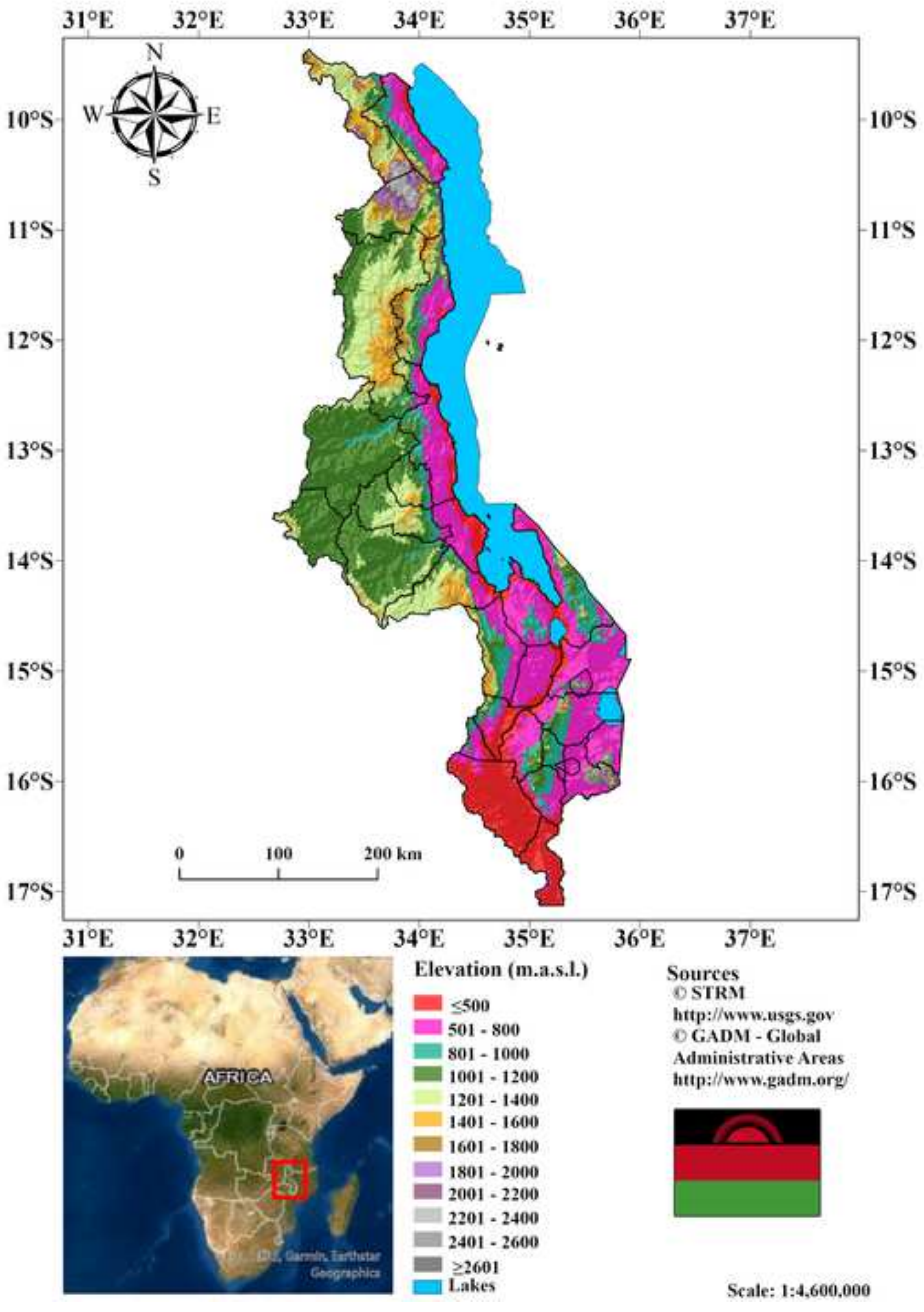

Figure 2

Click here to access/download;Figure;Temp and Prec.tif

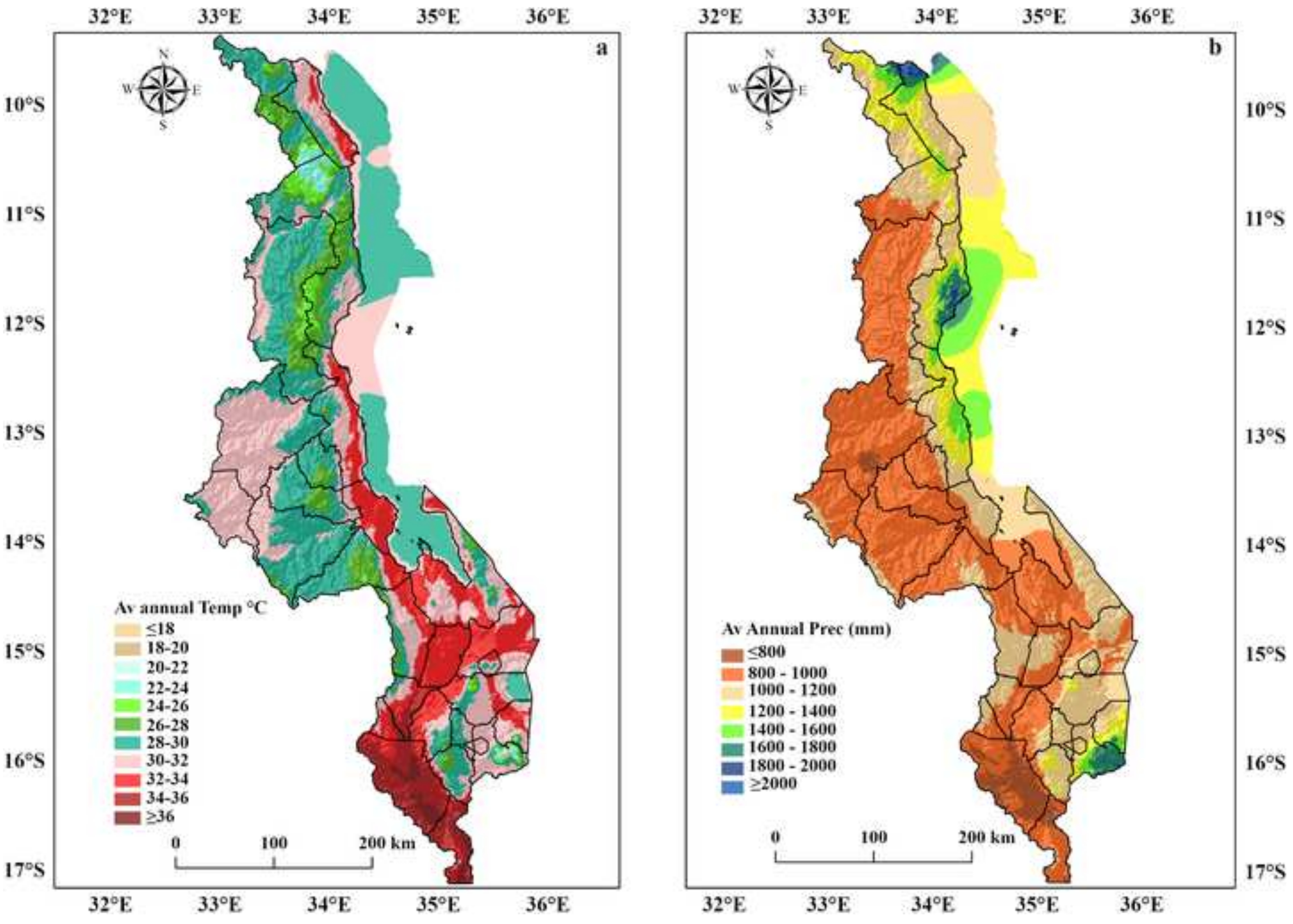

Figure 3 Click here to access/download;Figure;Presence points.tif

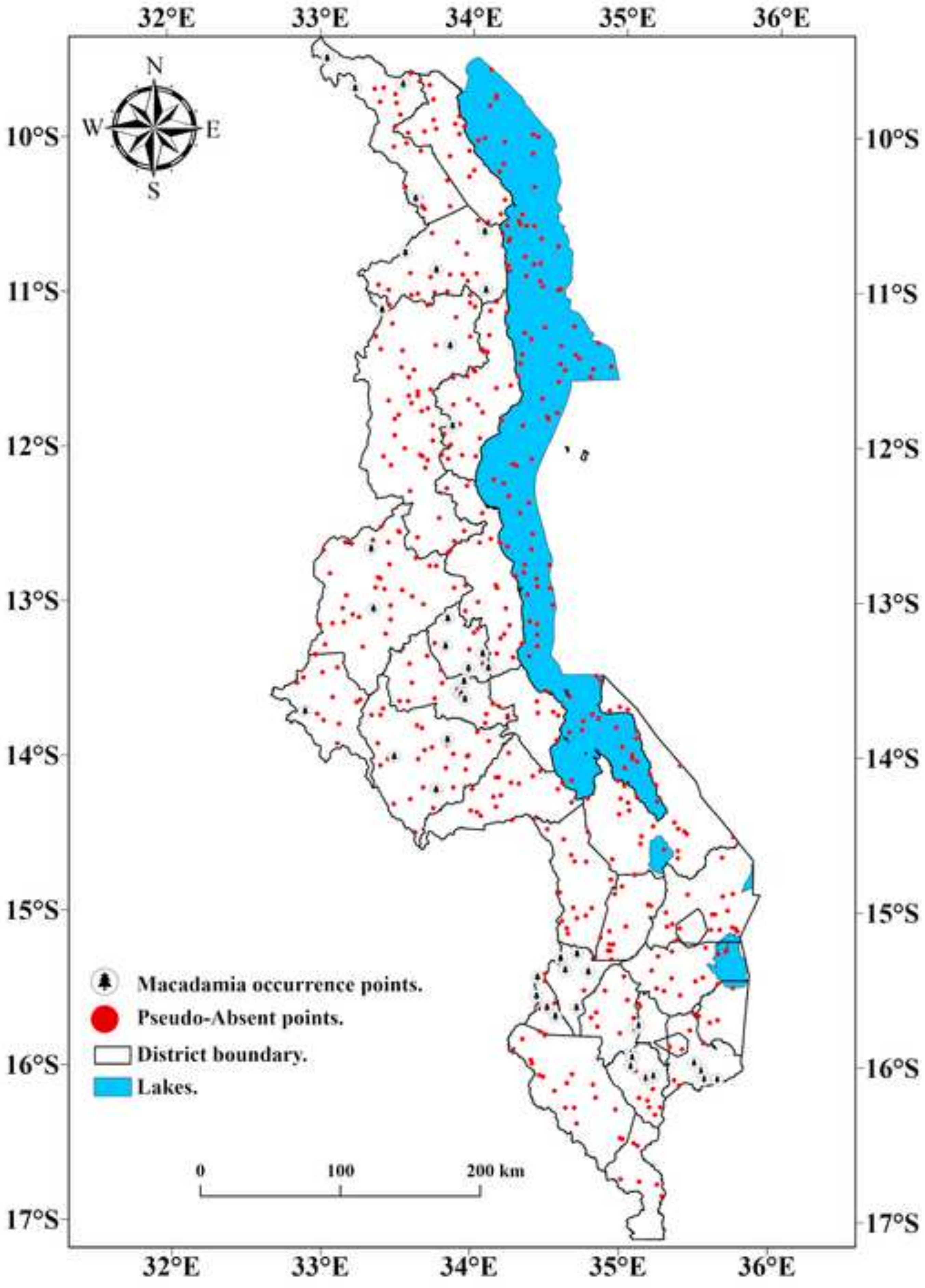

Figure 4

Click here to access/download;Figure;Curent_suit - Copy.tif

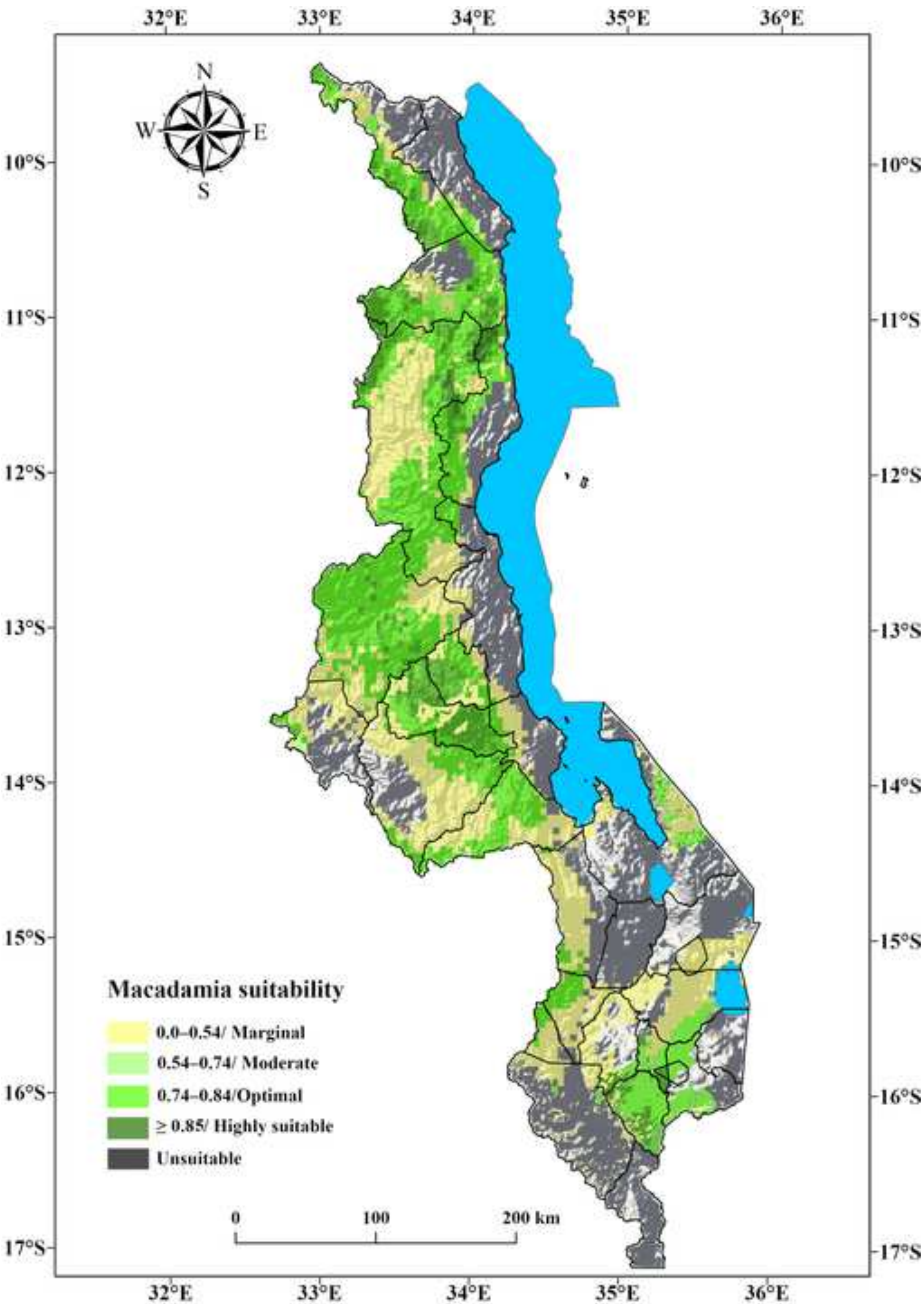

Figure 5

Click here to access/download;Figure;Changes in suitability.tif

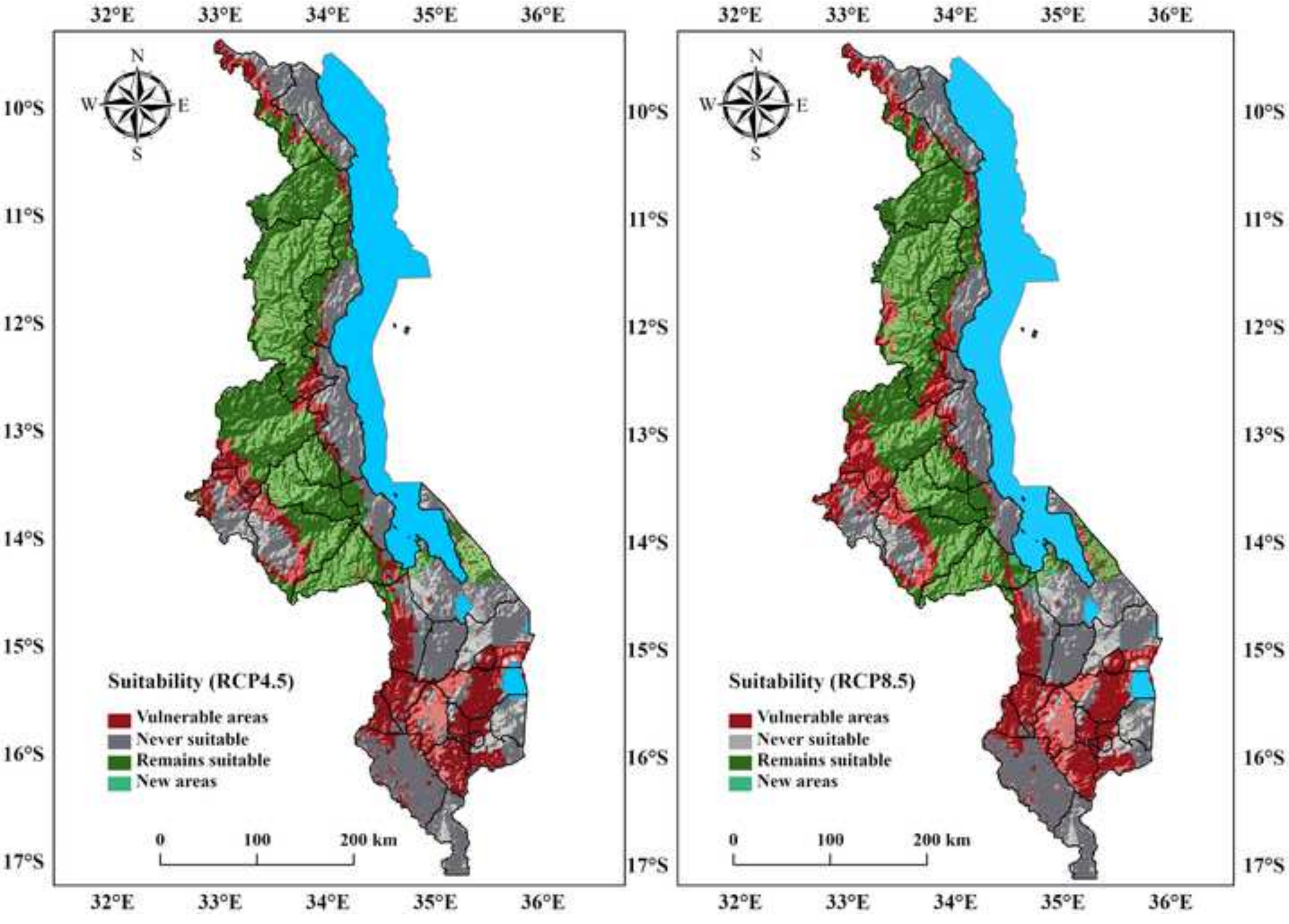

Supporting information

Click here to access/download
Supporting Information
Supplementary Information.pdf

Graphical abstract

Click here to access/download
Supporting Information
Graphical abstract done.pdf

Figures all

Click here to access/download
Supporting Information - Compressed/ZIP File Archive
Figures Final.rar
