# Supplementary material for: Climate suitability predictions for the cultivation of macadamia (Macadamia integrifolia) in Malawi using climate change scenarios
Source: PLoS One. 2021 Sep 9;16(9):e0257007. doi: 10.1371/journal.pone.0257007 (PMC8428786; doi:10.1371/journal.pone.0257007)
Supplement: Attachment — Submitted filename: Responses to the Editor and Reviewers.docx [file pone.0257007.s011.docx]

**Responses to the Editor**

1. The introduction which need to be streamlined to focus on the aim of the study.
   a. The introduction has been streamlined and we have made sure that it focusses on the aim of the study.
2. The choice of thresholds used in the study.
   a. The choice of thresholds in the study have been clarified in the methodology section. This was not properly explained in the first manuscript submission but we have corrected this mistake.
3. The authors should justify the choice of only 10 year old plantations, ensure that sampling bias correction of the presence points.
   a. We focus on ten-year-old macadamia orchards because the productivity of macadamia depends on the age of the orchard (i.e., the yield of the crop increases with age) (Barrueto et al., 2018) and at this age the crop is at the start of peak production. In order to make sure that we did not have sampling bias we randomly generated 500 pseudo-absence background points to act as neutral points (showing either the species is present or absent in an area).
4. Relate the variables to the crop being modelled (have agronomic rather than biophysical variables).
   a. This has been included in the discussion.
5. Ensure that the maps correspond to the suitable/unsuitable partitioning that was presented in the method section.
   a. We have included the thresholds that have been used to generate the five suitability classes in Fig 5. This was not initially included in the first submission.
6. Authors present VIF scores as variable importance metrics and this should be corrected and the results on variable importance appropriately presented and discussed.
   a. We have revised the results and included Fig 4 which shows the variable importance (%) explaining macadamia suitability in Malawi. This is based on an average of the 18 SDMs used in our analysis.
7. Scientific name of the crop can be added in the title.
   a. *Macadamia integrifolia* has been added in the title.
8. Line 17-19: the aim should capture both the current and future suitability modelling.
   a. This has been noted and addressed properly in the abstract.
9. Line 29: remove "the climate outputs", GCM always produce climate outputs and nothing else
   a. Done as advised.
10. Line 23-27: The results presented are conflicting, they need to be revised. The first sentence (Line 23) say that  "large parts of Malawi's macadamia growing regions will remain suitable for macadamia" but line 25 say that "suitable areas for macadamia production are predicted to shrink". The results need to be consistent and not confusing to the readers.
    a. We have revised the results and made sure that the confusion has been corrected
11. Line 29: Most? That is a qualitative result, probably based on visual assessments. Quantitative results are more conclusive.
    a. We have included the required percentages of the projected losses.
12. Line 55: Change will to may. Its on the balance of probability.
    a. We have revised the introduction and this was taken into account.
13. Line 75-93: This information may not be necessary in the introduction. Be succinct and focused on the aim of the study and avoid broad issues
    a. This has been removed from the introduction as it was rendered no longer important.
14. Line 99-100:: 98-100: Yes indeed, macadamia could have been omitted but that is not sufficient motivation for the study. Authors to clearly state the gap they are filling with their study (especially in light of the work by Evans, N. (2008). Suitability Mapping of the Malawi macadamia industry. *Irish Aid: Dublin, Ireland*). There is need for this study but it is not well articulated in the introduction.
    a. The introduction has been re-written to make sure that it reflects the aim of the study.
15. Line 118: Detailed and summary are conflicting words, it is not possible to have a detailed summary, a summary is a short version of the detailed report. it is also not a good idea to have reference to Supplementary materials in the Introduction section.
    a. Edited and removed from the introduction.
16. Line 120: It is an important point that the perennial crops also store carbon and therefore an important aspect in climate mitigation. This important aspect is not well articulated in the introduction and just appears hanging here. You can further explain on this important aspect of the suitability study as part of your justification/motivation.
    a. Done.
17. Line 127: It will be good to indicate who and how the results can be used in climate planning.
    a. We have included this sentence “In addition to informing policy and trade, this assessment is a first step toward identifying and implementing adaptation measures tailored to macadamia within global boundaries”.
18. Line 127: Overall introduction: The introduction is too long, winding and need to be more focused.
    a. The introduction has been shorted and provides more clarity on the aim of the study.
19. Line 157: Since Malawi is not on the equator, what is the equivalent in Malawi?
    a. To ensure that we did not bring in more confusion in the methodology we have changed the sentence.
20. Line 162: RCP4.5 is not the optimistic scenario, the optimistic scenario is RCP2.6, which you did not use. it is important to be consistent so that we avoid confusing readers.
    a. Edited and made sure that this is clear.
21. Line 165: 2050s (period 2046–2065): Kindly recheck if this is correct, it could be 2040-2060.
    a. 2040-2060 is the correct one.
22. Line 187: Justify the choice of the 0.77 threshold.
    a. This has been addressed and is based on past research work on agroforestry modelling. Has been highlighted in the revised manuscript.
23. Line 193: "Hence, absence data may not represent naturally occurring phenomena" This is not clear, revise.
    a. This has been revised.
24. Line 196: 500 randomly generated pseudo-absence.
    a. We generated 500 random pseudo-absence points considering the area of Malawi. We did not use the conversional 10,000 pseudo-absence points because this is mainly for global studies and not country studies i.e. global is bigger and country level is small.
25. Line 221: The criteria to assess GCMs is to measure their performance on historical measurements and see which GCM is able to capture the trends and rhythms of the data.
    a. This has been noted and we have revised our sentence on this.
26. Line 227: Section 3.1: Authors should recheck the results as they appear to be from VIF and not variable importance. The results and related discussion should therefore be revised accordingly.
    a. Explained above.
27. Table 1: Move to Methods (and also add at the end of the manuscript and not inside text.
    a. Done.
28. Line 242: The plants will most likely not be able to grow on water, so the percentage of ONLY land area is more representative.
    a. We present total land surface area and not the lake. The 53,925 km^2^ is based from the 94, 449 km^2^ of land excluding the lake.
29. Line 243-249: The descriptions of the colours should be below the Figure and not in the results. In addition these results are confusing because in the methods the authors say they used one threshold to get suitable and unsuitable areas but here they have 5 suitability classes. They should stick with their two classes whose methods are explained or explain in the methods section how the categories were generated. In addition, the authors present the results in terms of elevation but this is not one of the variables used in the modelling. All results using the 5 classes are therefore difficult to comprehend and understand if the choice of the classes are not scientifically justified.
    a. Resolved.
30. Line 247-251. The sentences are conflicting and should be revised. First authors claim that optimal suitability is in areas not exceeding 30 degrees (line 249) and then went on to mention that some optimal suitable areas are in areas above 30 degrees (line 251). This should be revised.
    a. We have made the sentences more clear.
31. Line 259: Remove "Though, these areas are also used for the production of other crops, particularly annuals". The results sections should be reserved to the results of the study and not anything else.
    a. Done.
32. Line 267-268: This is now discussion and should be moved to the discussion section.
    a. It has been removed.
33. Line 270: " significant reductions" this is qualitative (and subjective), quantitative results are more convincing, what is the loss in suitability in the district, in numbers.
    a. Included the loss which is 100% of the area.
34. Line 271-273: Leave that for the discussion section.
    a. Removed from the results.
35. Line 280: RCP4.5 is not the optimistic scenario.
    a. Revised.
36. Line 283-284: Not clear, revise.
    a. Rephrased to make sure that it is clear.
37. Line 284-285: Explanations should be reserved for discussion section.
    a. Moved to discussion.
38. Line 293-317: The authors should recheck the variable importance results and revise this section accordingly.
    a. Check as above
39. Line 332-334: Revise the statement for clarity/sense.
    a. Revised
40. Line 335 - 343: It will be important if the authors discuss their results with regard to how the impacts are likely to occur on the crop. for example which phenological stage is likely to be most affected and what is the effect of increased temperature on growth and production capacity of the plants, what happens when water is not enough or when water is not supplied during flowing. This makes the scientific basis of the study strong.
    a. This has been taken into account.

**Responses to Reviewer #1**

- I am little sceptical about the criteria used for selection of districts and representativeness of the 120 orchards sampled. The close proximity of orchards in some districts (Fig 3) is troublesome for me.
  - Thank you for noting this. In the first place Fig 3 shows the data that was collected from the field. However, we resampled the occurrence points to a tolerance of 5 km so that no two points could be found in one environmental layer at a resolution of 5 km x 5 km. This was not initially well presented but has been rephrased.
- Authors sampled 10-year old macadamia orchards yet they do not provide the justification for this decision. As it looks, exclusion of <10 and >10 year orchards introduces bias in the dataset. My argument is that an orchard is an orchard irrespective of how old. I now wonder if using pseudo-absence points was to conceal this sampling limitation.
  - The response to this is highlighted in the above number 3.

**Responses to Reviewer #2**

- The authors need to structure the flow of ideas, concepts and motivation in the introduction section where few redundant ideas were observed.
  - We have revised the introduction and made sure that there is proper flow of ideas. Thanks for the recommendations.
- The authors must justify why they used the RCP 4.5 and 8.5 and not any of the other scenarios.
  - This has been justified accordingly in the revised manuscript.
- Line 196: Provide a statistical justification of the use of 500 background pseudo-absence points and not the widely used 10 000 points.
  - We utilized 500 background pseudo-absence points because of the size of Malawi. Usually the 10 000 points are used for global or regional studies. We based our decision following Barbet-Massin et al. (2012).
- Authors must expand and clarify on the 17 GCMs used enough to be repeatable. Which ones were used and why.
  - As highlighted in the methodology we utilized results from the average rather than single GCMs results for this analysis. This was done in order to reduce inherent uncertainties from individual GCM results.
